# The node of Ranvier influences the *in vivo* axonal transport of mitochondria and signaling endosomes

**DOI:** 10.1016/j.isci.2024.111158

**Published:** 2024-10-11

**Authors:** Andrew P. Tosolini, Federico Abatecola, Samuele Negro, James N. Sleigh, Giampietro Schiavo

**Affiliations:** 1 Department of Neuromuscular Diseases and UCL Queen Square Motor Neuron Disease Centre, Queen Square Institute of Neurology, University College London, London WC1N 3BG, UK; 2 School of Biomedical Sciences, Faculty of Medicine, The University of Queensland, St Lucia, QLD 4067, Australia; 3 Australian Institute for Bioengineering and Nanotechnology, The University of Queensland, St Lucia, QLD 4067, Australia; 4 Department of Biomedical Sciences, University of Padua, 35131 Padua, Italy; 5 U.O.C. Clinica Neurologica, Azienda Ospedale, University of Padua, 35128 Padua, Italy; 6 UK Dementia Research Institute, University College London, London WC1E 6BT, UK

**Keywords:** Molecular neuroscience, Cellular neuroscience

## Abstract

Efficient long-range axonal transport is essential for maintaining neuronal function, and perturbations in this process underlie severe neurological diseases. Nodes of Ranvier (NoR) are short, specialized unmyelinated axonal domains with a unique molecular and structural composition. Currently, it remains unresolved how the distinct molecular structures of the NoR impact axonal transport dynamics. Using intravital time-lapse microscopy of sciatic nerves in live, anesthetized mice, we reveal (1) similar morphologies of the NoR in fast and slow motor axons, (2) signaling endosomes and mitochondria accumulate specifically at the distal node, and (3) unique axonal transport profiles of signaling endosomes and mitochondria transiting through the NoR. Collectively, these findings provide important insights into the fundamental physiology of peripheral nerve axons, motor neuron subtypes, and diverse organelle dynamics at the NoR. Furthermore, this work has relevance for several pathologies affecting peripheral nerves and the NoR.

## Highlights

• Fast and slow motor neurons exhibit similar NoR morphology *in vivo*
• Signaling endosomes and mitochondria accumulate specifically at the distal NoR
• Transport dynamics of both organelles are altered at the NoR
• Fastest transport speeds were observed at the internode proximal to the NoR

## Graphical abstract

**Figure:**
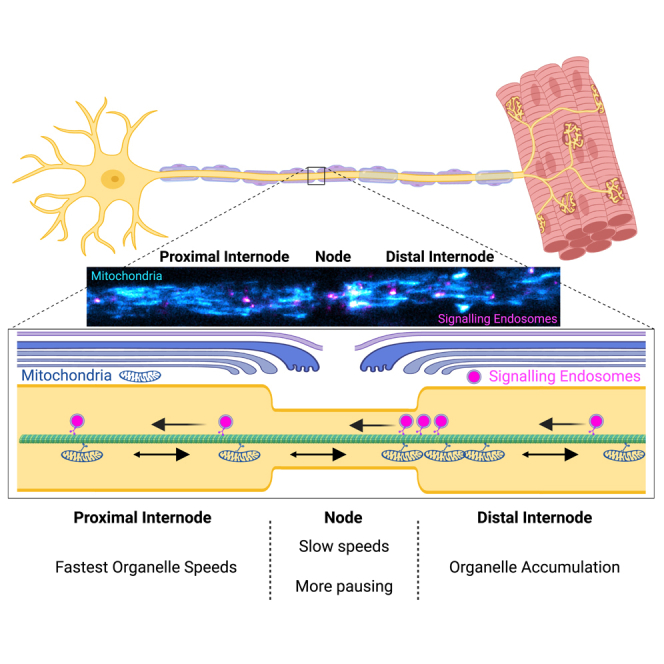

## Introduction

Axonal transport is a fundamental biological process that maintains neuronal homeostasis with constant bidirectional shuttling of essential cargoes and structural components between the neuronal cell body and axon termini. Along the microtubule network, cytoskeletal elements (e.g., neurofilaments), as well as membranous (e.g., mitochondria, endosomes) and membrane-less (e.g., RNA granules) organelles undergo plus-end directed anterograde transport driven by members of the kinesin motor protein family, and minus-end directed retrograde transport via cytoplasmic dynein.^1^^,^^2^ Effective long-range axonal transport is essential for maintaining neuronal function, and trafficking perturbations underlie several neurodevelopmental and neurological conditions.^3^

α-motor neurons (MNs) can be subclassified into fast MNs (FMNs) and slow MNs (SMNs) that selectively innervate specific skeletal muscle fiber subgroups. Both types of MN and the muscle fibers they innervate have distinct metabolic, functional, and transcriptional properties.^4^^,^^5^ Compared to SMNs that innervate slow oxidative type I muscle fibers, FMNs have larger motor unit sizes, faster nerve conduction, and different firing patterns, and they innervate type IIa fast oxidative-glycolytic, as well as type IIx and IIb fast glycolytic muscle fibers.^4^^,^^6^

We have previously determined that signaling endosome transport speeds are similar between motor axons innervating prototypical fast and slow muscles.^7^ Likewise, axonal transport speeds are comparable between hindlimb and forelimb peripheral nerves.^8^ In contrast, signaling endosome transport in MNs is faster than in sensory neurons innervating the same muscle.^9^ Several factors directly influence axonal transport dynamics,^1^^,^^10^^,^^11^^,^^12^ and can be perturbed in disease.^3^^,^^13^ Collectively, this suggests that the axonal transport machinery is differentially modulated in physiological and pathological contexts.

As most *in vivo* axonal transport studies have prioritized larger and/or consistently sized axonal segments, our understanding of transport dynamics at highly specialized axonal regions, such as the node of Ranvier (NoR), remains incomplete. NoRs are short uncovered axonal domains that facilitate action potential propagation, and each of the four regions (i.e., node, paranode, juxtaparanode, and internode) is comprised of distinct structural and functional proteins.^14^^,^^15^ Electron microscopy reveals reductions in axonal diameters at the NoR, which maximize electrical conduction velocities^16^; these constrictions were attributed to reduced neurofilament, but not microtubule, content.^17^^,^^18^^,^^19^ Most internodal neurofilaments are not continuous through the NoR and cease near the juxtaparanode, whereas microtubules extend through the NoR to connect adjacent internodes,^20^ providing structural continuity for the continuous processive movement of cargoes.

Despite high organelle content,^18^^,^^21^ unique axonal Ca^2+^ dynamics^22^ and high metabolic needs,^23^ which are all features with the potential to regulate axonal transport, few studies have investigated transport dynamics at the NoR. Neurofilaments undergoing slow axonal transport^24^ increase their speed through the NoR.^25^^,^^26^ In contrast, muscle-administered horseradish peroxidase (HRP),^27^ radiolabeled glycoproteins,^28^ and lysosome-linked enzymatic activity^29^ are enriched at peripheral nerve NoRs, but little is known about their transport.

The aim of this study was to assess *in vivo* axonal transport of two different organelles at the NoR in FMNs and SMNs to further our understanding of fast axonal transport dynamics through this specialized structure. Knowledge of how the node affects cargo delivery may reveal key mechanisms relevant to pathologies affecting peripheral nerves.

## Results

### Identifying the node of Ranvier *in vivo*

To confirm the identity of the NoR, we performed immunohistochemistry on fixed, teased sciatic nerve fibers from ChAT.eGFP mice probing for established markers of the node and paranode.^30^ As expected, we observed a ring-shaped cluster of Na_V_1.6 channels approximately in the middle of the ChAT.eGFP nodal constriction that is flanked by CASPR immunolabelling of the paranode (Video S1). Therefore, we concluded that the identical constrictions observed in ChAT.eGFP sciatic nerves contain the nodal and paranodal regions and can be distinguished from the larger internodal regions using intravital imaging of the endogenous eGFP fluorescence in ChAT.eGFP mice.

**Figure 1:**
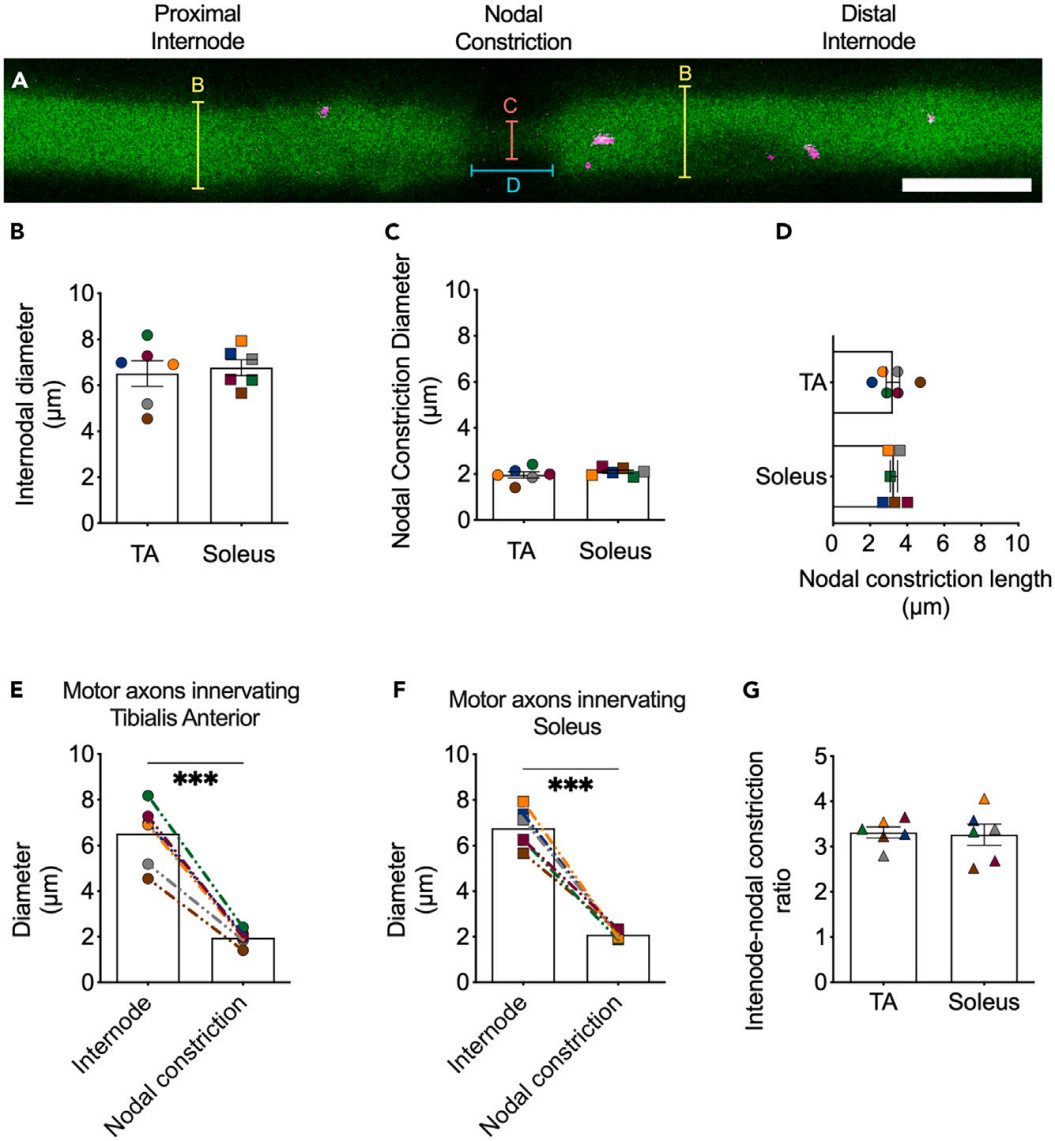
Nodal morphology is similar between fast and slow motor axons of the sciatic nerve (A) Representative single frame image of H_C_T-555 containing signaling endosomes (magenta) in a single *in vivo* motor axon (green) from a ChAT.eGFP mouse sciatic nerve motor axon. Scale bar = 5 μm. Yellow lines represent the axonal region assessed to quantify the internodal diameters in (B), the orange line represents the axonal region assessed to quantify the nodal constriction in (C), and the cyan line represents the axonal region assessed to quantify the length of the nodal constrictions in (D). Motor axons innervating tibialis anterior (TA) or soleus muscles display similar (B) internodal diameters (*p* = 0.71, unpaired two-tailed *t*-test), (C) nodal constriction diameters (*p* = 0.40, unpaired two-tailed *t*-test), and (D) nodal constriction lengths (*p* = 0.90, unpaired two-tailed *t*-test). Furthermore, the differences in axonal diameters at the internode and nodal constriction were comparable between motor axons innervating (E) TA and (F) soleus. (G) The ratios of internodal to nodal diameters were also comparable. (B, C, D, and G) were assessed by an unpaired, two-tailed t test. (E and F), were assessed by a paired two-tailed t-test (∗∗∗*p* < 0.001). For all graphs, *n* = 6, data are represented as mean ± SEM, and the color-coding remains consistent between animals. Linked with Video S4.

Video S1. Na_V_1.6 clusters are located in the middle of the nodal constriction and are flanked by CASPR-positive paranodal regions in a peripheral nerve motor axon – linked to Figure 1 Representative videos of (A) a rotating max projection z-stack, and (B) slice-by-slice through the z-axis, highlighting the (i) cholinergic (i.e., ChAT-positive) motor axon, the immunolabeled (ii) nodal (Na_V_1.6) and (iii) paranodal (CASPR) regions, along with the (iv) overlay, from teased axons of a ChAT.eGFP mouse sciatic nerve. Scale bar = 5 μm.

To locate the NoR in FMN and SMN axons *in vivo*, we performed intramuscular injections of H_C_T separately into TA or soleus muscles in ChAT.eGFP mice. Owing to the divergent muscle composition, the TA was selected to assess transport in FMN axons, whereas the soleus was chosen to assess transport in SMN axons. Indeed, TA and soleus are prototypical fast and slow muscles, respectively, with the TA being comprised of ∼45–60% type IIb, ∼30–45% type IIx, ∼10–20% type IIa and ∼0–3% type I muscle fibers, and the soleus consisting of ∼40% type I, ∼40% type IIa and ∼20% type IIx muscle fibers.^31^^,^^32^ H_C_T is internalized into axon termini of motor neurons through binding to polysialogangliosides, nidogens,^33^ and the tyrosine phosphatases, LAR and PTPRδ.^34^ Following sorting into Rab5-positive endosomal compartments, H_C_T undergoes fast retrograde axonal transport toward the cell bodies of spinal cord motor neurons in Rab7-positive organelles^35^^,^^36^ using a cytoplasmic dynein- and microtubule-dependent process.^37^ Therefore, H_C_T injections simultaneously enabled *in vivo* assessments of axonal signaling endosomes and the labeling of motor neurons innervating distinct muscles.

Intravital imaging of ChAT.eGFP sciatic nerve cholinergic (i.e., motor) axons enabled the identification of nodal constrictions bordered on both sides by wider internodal regions (Figure 1A). Assessing individual TA-innervated FMN or soleus-innervated SMN axons revealed similar diameters of the internodal regions (Figure 1B) and nodal constrictions (Figure 1C), as well as lengths of the nodal constrictions (Figure 1D). This analysis also revealed that the differences in axonal diameters at the internode and nodal constriction were comparable between motor axons innervating TA (Figure 1E) and soleus (Figure 1F) muscles, which was confirmed when the ratios of internodal to nodal diameters were calculated (Figure 1G). From these analyses, we concluded that morphological features of the NoR are equivalent in wild-type motor axons innervating prototypical fast and slow muscles. Furthermore, nodal morphology in TA-innervating motor axons is similar between ChAT.eGFP and Mito.CFP mice (Figure S1), which are the main two transgenic fluorescent strains used in this study.

### Signaling endosomes and mitochondria cluster at the distal Nodes of Ranvier

**Figure 2:**
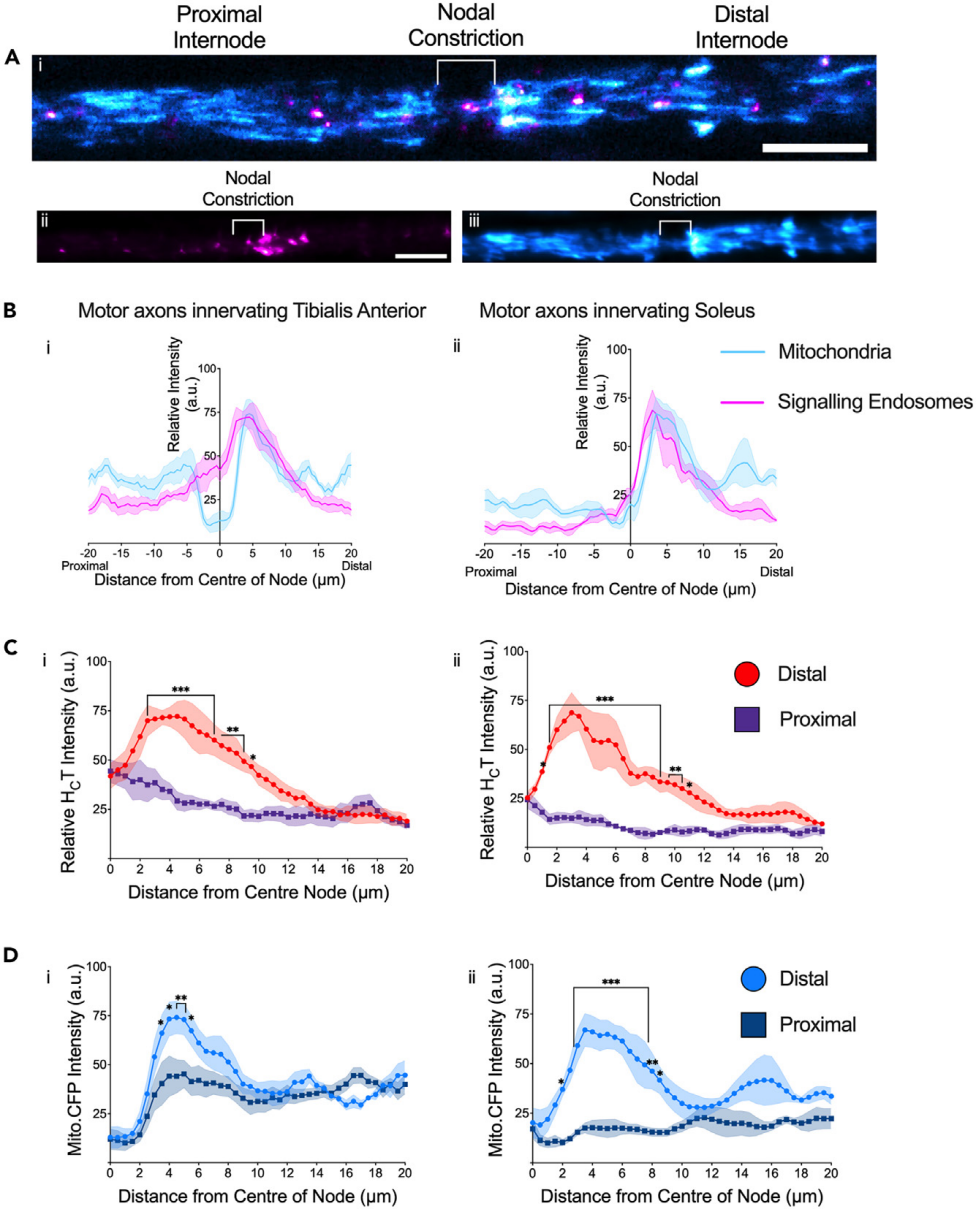
Signaling endosomes and mitochondria selectively accumulate at sites distal to the node of Ranvier (A) (i) Representative image demonstrating the spatial distribution of H_C_T-containing signaling endosomes (magenta) and mitochondria (cyan) from a single tibialis anterior-innervating motor axon in a Mito.CFP mouse. Representative z stack projection (sum of the slices) from a time-lapse video (see Video S2) indicates that the fluorescence profiles of (ii) H_C_T-containing signaling endosomes and (iii) Mito.CFP-labeled mitochondria are enriched on the distal side of the NoR. Scale bar = 10 μm. (B) In motor axons innervating the (i) tibialis anterior and (ii) soleus muscles, we observed increased average relative fluorescence intensities of both H_C_T-containing signaling endosomes (magenta) and Mito.CFP-labeled mitochondria (cyan), exclusively on the distal side of the NoR. Enhanced fluorescence profiles of (C) H_C_T-containing signaling endosomes between 1 and 11 μm from the center of the NoR (i) *TA* - Axonal Location: *p* < 0.001; Mean Relative Fluorescence: *p* < 0.001; Interaction: *p* < 0.001. (ii) *Soleus:* Axonal Location: *p* < 0.001; Mean Relative Fluorescence: *p* < 0.001; Interaction: *p* < 0.001), and (D) mitochondria 2–8.5 μm from the center of the NoR (i) *TA* - Axonal Location: *p* < 0.001; Mean Relative Fluorescence: *p* < 0.001; Interaction: *p* = 0.0002. (ii) *Soleus -* Axonal Location: *p* < 0.001; Mean Relative Fluorescence: *p* < 0.001; Interaction: *p* < 0.001). For all graphs, *n* = 5 animals, 16 axons, data are represented as mean (solid line) ± SEM (dashed lines). (C and D) Data were compared by two-way ANOVA and Šídák’s multiple comparisons tests (∗*p* < 0.05, ∗∗*p* < 0.01, ∗∗∗*p* < 0.001). See also Figures S1 and S2.

We next profiled the spatial location of signaling endosomes and mitochondria in relation to the nodal constriction in fast and slow motor axons. To label the different motor axon subtypes, we injected H_C_T into either TA or soleus muscles of Mito.CFP mice, and performed simultaneous time-lapse microscopy of H_C_T-containing signaling endosomes and mitochondria,^38^ focusing specifically on the NoR (Figure 2Ai; Video S2). The individual fluorescence intensity profiles of both organelles were first created using z-projections of individual frames from time-lapse videos (Figure 2Aii-iii), and then the fluorescence intensities of signaling endosomes and mitochondria were assessed across the whole region (80 μm). We detected an accumulation of both organelles specifically at the distal side of the nodal region (Figure 2B). To quantify this, we directly compared the average axonal fluorescence intensities of signaling endosomes and mitochondria from individual videos of TA- and soleus-innervating axons from proximal and distal internodal regions. These analyses revealed that there were a greater number of immobile H_C_T-containing signaling endosomes (Figure 2C) and mitochondria (Figure 2D) at the distal side of the NoR, and this distribution was similar in TA- and soleus-innervating motor axons (Figures 2C and 2D). To rule out that this clustering phenotype was caused by the administration of H_C_T and generation of H_C_T-containing signaling endosomes *in situ*, we evaluated the mitochondrial fluorescence profiles in naive Mito.CFP axons (i.e., without intramuscular H_C_T injections). This analysis revealed the same mitochondrial distribution as shown in Figure 2D, demonstrating that the accumulation of mitochondria seen distally at the NoR is independent of H_C_T (Figure S2).

**Figure 3:**
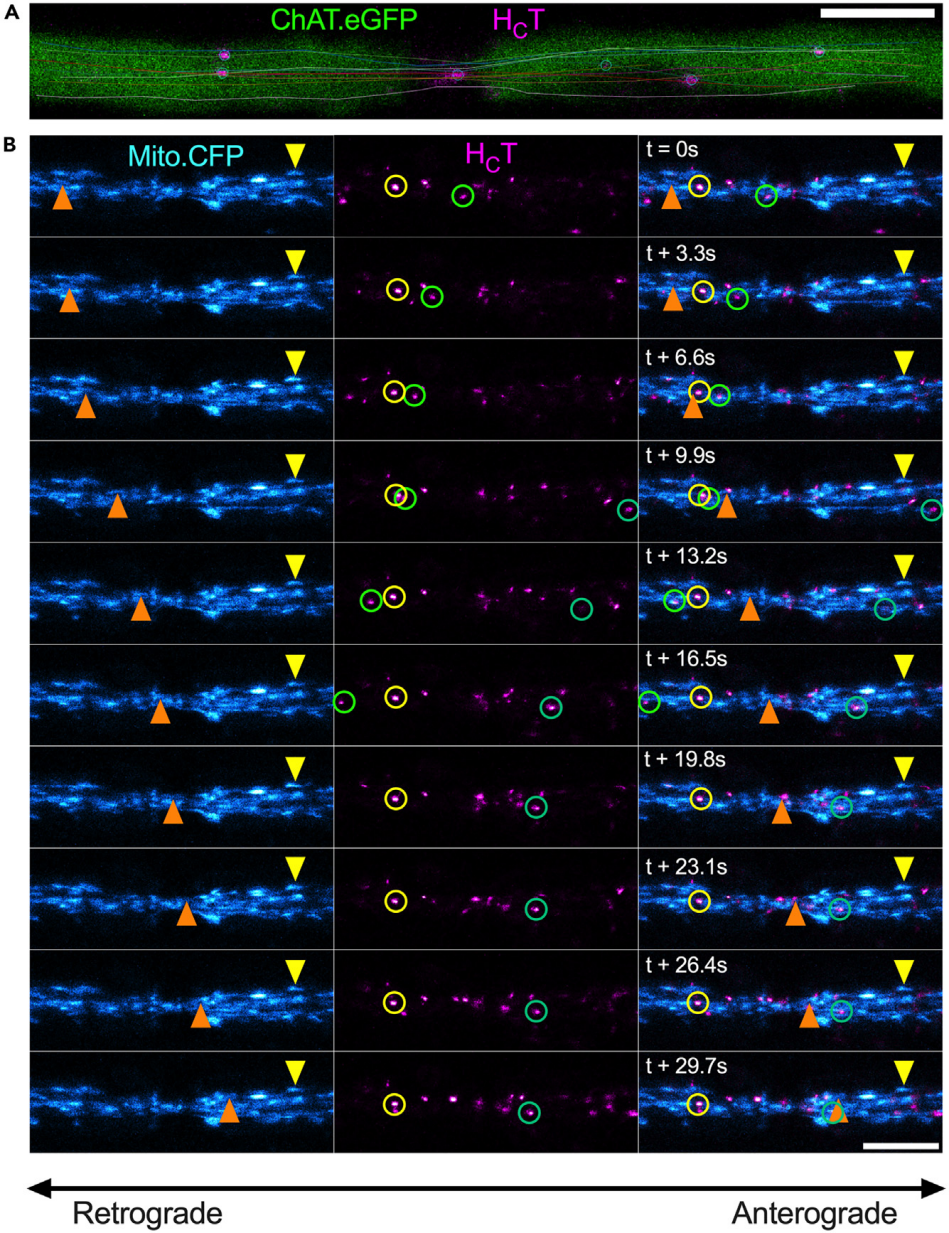
Axonal cargoes are bi-directionally funnelled through the node of Ranvier (A) *In vivo* axonal transport analysis at the node of Ranvier (NoR) reveals that H_C_T-containing signaling endosomes (circles) narrow their trajectories (lines) from the wider intranodal regions to the cytoskeletal confines at the nodal constriction. Representative image of a sciatic nerve motor axon from a ChAT.eGFP mouse. (B) Time-lapse images taken every 3.3 s of *in vivo* axonal transport of Mito.CFP-labeled mitochondria (cyan) and H_C_T-containing signaling endosomes (magenta) specifically focused at the NoR. Green circles identify retrogradely moving signaling endosomes, orange triangles identify an anterogradely moving mitochondrion, and yellow triangles/circles identify paused/immobile organelles. Anterograde movement is from left to right, and retrograde movement is in the opposite direction. Scale bars = 5 μm.

Video S2. *In vivo* axonal transport of mitochondria and signaling endosomes is observed through the nodal constrictions of a single sciatic nerve axon – linked to Figures 2-3 Representative time-lapse microscopy video of dual-organelle *in vivo* axonal transport through the nodal constriction of a Mito.CFP mouse. Frame interval = 1.44 s, number of frames: 201, playback rate: 15 fps, scale bar = 10 μm.

Previous studies suggest that axon-Schwann cell interactions regulate several critical functions. For example, CXCL12α/SDF-1 released from perisynaptic Schwann cells promotes motor axon regeneration,^39^ and ATP released from neurons activates Schwann cells.^40^ We, therefore, aimed to evaluate whether H_C_T-positive signaling endosomes are released by exocytosis from this site of communication between motor axons and Schwann cells. To do so, we injected H_C_T into TA muscles in the PLP-GFP mouse, in which GFP is exclusively expressed in Schwann cells.^41^ 24 h after H_C_T injections, teased individual axons were isolated from the PLP-GFP sciatic nerves, and imaged. H_C_T clusters were again found in the distal portion of the NoR; however, no signaling endosomes were detected in the cytosol of Schwann cells (Video S3), ruling out that H_C_T is transferred from motor axons to Schwann cells. Altogether, using three separate transgenic reporter mouse models, we have shown that H_C_T-positive signaling endosomes, along with mitochondria, cluster specifically at the distal side of the NoR.

Video S3. Signaling endosomes that traverse the nodal constrictions are not present in the Schwann cell cytosol Representative video of a (A) rotating max projection z-stack, and (B) slice-by-slice through the z-axis, highlighting the (i) H_C_T-containing signaling endosomes (magenta), within the (ii) Schwann cell cytosol (eGFP), and (iii) overlay, from the same teased axon of a PLP-eGFP mice sciatic nerve. Scale bar = 10 μm.

### Axonal transport of signaling endosomes and mitochondria through the node of Ranvier

**Figure 4:**
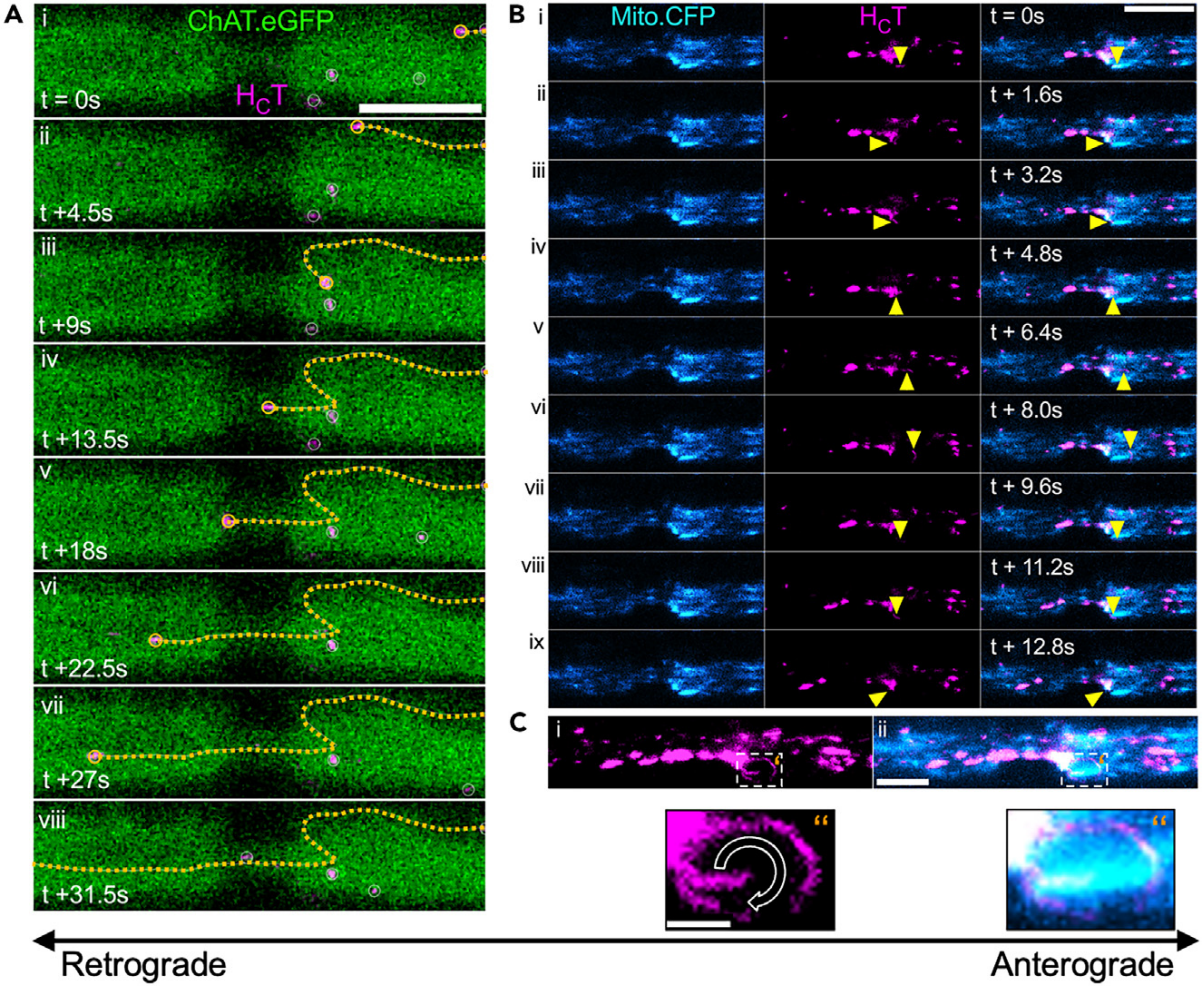
Non-linear trajectories of signaling endosomes during their transit through a node of Ranvier (A) Time lapse image series of an individual H_C_T-containing signaling endosome (magenta, with orange circle) traveling on an “S-shaped” trajectory (orange dotted lines) during *in vivo* axonal transport through the NoR from a ChAT.eGFP sciatic nerve motor axon. Frame interval = 4.5 s; scale bar = 10 μm. Linked with Video S5. (B) Time lapse image series demonstrating an individual H_C_T-containing signaling endosome (magenta, with yellow arrowheads) traversing in a “circular” pathway (contained inside the white dashed lines) around an individual mitochondrion (cyan) on the distal side of the NoR in a Mito.CFP sciatic nerve axon. Frame interval = 1.6 s; scale bar = 10 μm. Linked with Video S6. (C) z stack maximum projection of (B), highlighting the circular pathway exhibited by an individual signaling endosome (magenta) as it traverses around a mitochondrion. C″ are enlargements of the boxed areas in C’. Scale bar: C = 10 μm, Scale bar: C’’ = 2 μm.

We next sought to determine the axonal transport dynamics of both signaling endosomes and mitochondria through the NoR. First, we established that in ChAT.eGFP motor axons, H_C_T-containing signaling endosomes narrow their trajectories as they traverse the nodal constriction (Figure 3A; Video S4). We found that bidirectionally moving mitochondria also funnel their paths through the NoR when transitioning from the larger internodal regions to the nodal constriction, and then widen their trajectories when entering the subsequent internodal region (Figure 3B; Videos S2 and S4). We also observed non-linear courses that individual signaling endosomes would take as they approached the nodal constriction (e.g., moving in an “S” shape (Figure 4A; Video S5) or traveling in a circular motion (Figures 4B and 4C; Video S6)). These unusual trajectories might be caused by signaling endosomes traversing areas where the normal microtubule distribution present in internodal regions is altered due to proximity to nodal constrictions, e.g., radial axonal expansions,^42^ or circling areas with a high density of immobile organelles (e.g., mitochondria, Figure 4C).

**Figure 5:**
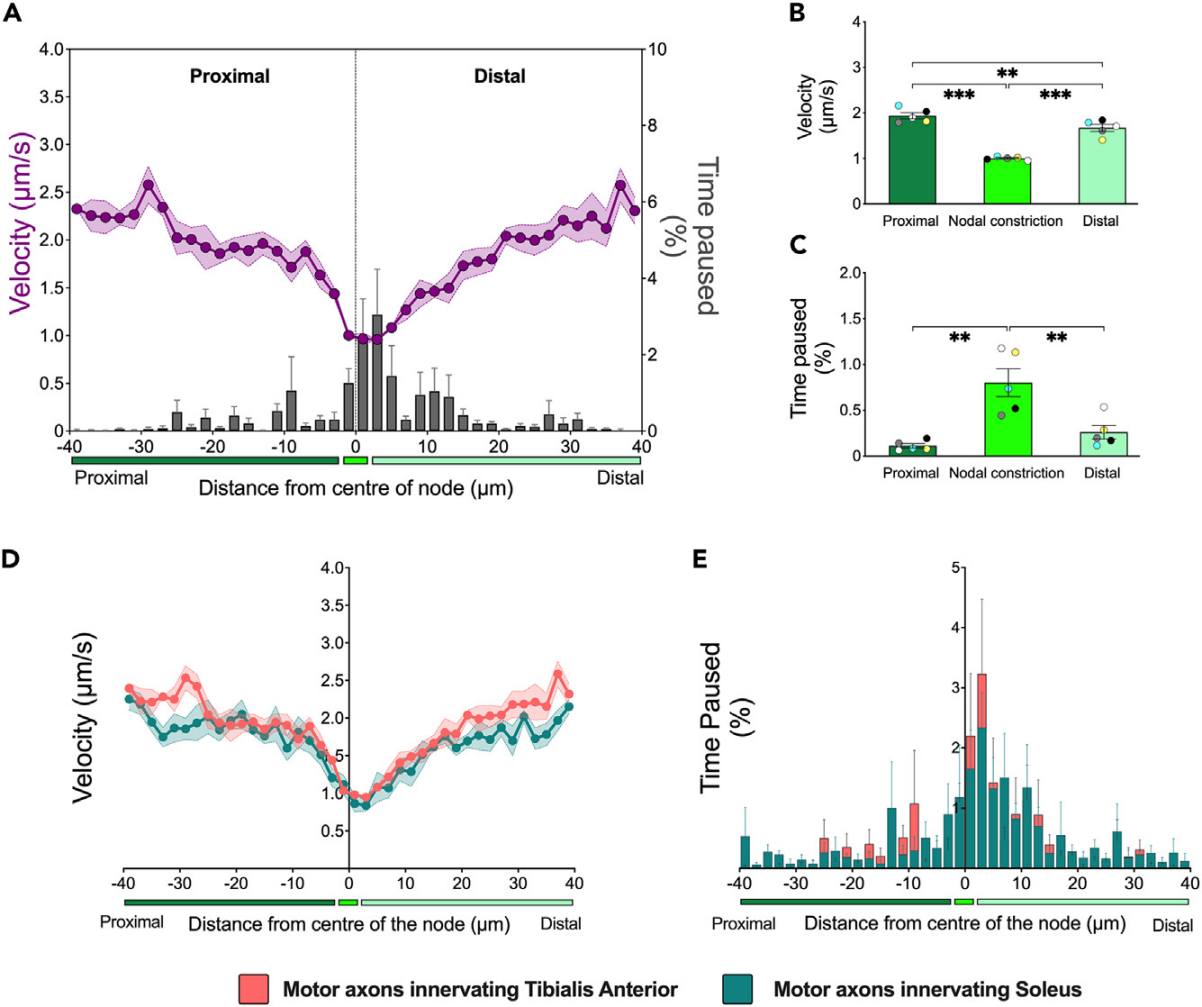
Signaling endosomes decelerate and pause more at nodes of Ranvier regardless of motor neuron subtype (A) Retrograde axonal transport dynamics (mean moving velocity [purple] and relative frequency of mean pausing [gray bars]) of H_C_T-containing signaling endosomes plotted across the nodal constriction and beyond (80 μm distance) in motor axons innervating the tibialis anterior muscle. The x axis represents the distance from the center of the node of Ranvier (μm) and is split into three segments: (1) *Proximal* = 38 μm of the proximal internode (dark green); (2) *Center* = 4 μm representing the mean nodal length, (as determined in Figure 1D; bright green); and (3) *Distal* = 38 μm of the distal internode (light green). Comparisons across the proximal internode, nodal constriction and distal internode of the (B) mean moving velocity (*p* < 0.001), and (C) relative frequency of mean time paused (*p* < 0.001). (B and C) Data were compared by one-way ANOVA, followed by Holm-Šídák’s multiple comparisons test. Histograms comparing the (D) mean moving velocity and (E) relative frequency of mean time paused from motor axons innervating the tibialis anterior (salmon) and soleus (teal). For all graphs, data are represented as mean (solid line) ± SEM (dashed lines/error bars), *n* = 4 (soleus) and *n* = 5 (tibialis anterior) from Mito.CFP mice, ∗∗*p* < 0.01, ∗∗∗*p* < 0.001. See also Figures S3 and S5.

Video S4. Representative video of *in vivo* axonal transport of H_C_T-containing signaling endosomes traversing the node of Ranvier in a single sciatic motor axon – Linked to Figures 3-5 Top panel = ChAT.eGFP signal; Middle panel = retrogradely transporting H_C_T-containing signaling endosomes; Bottom panel = overlay. Frame interval: 0.53 s, number of frames: 400, frames per second: 20, scale bar = 10 μm.

Video S5. Retrograde axonal transport of H_C_T (magenta) can display an unusual trajectory through the node of Ranvier in peripheral nerve motor axons (green) – Linked to Figure 4A Frame interval: 0.45 s, playback rate = 25 fps, acquisition time = 41 s, scale bar: 10 μm.

Video S6. Retrograde axonal transport of H_C_T-containing signaling endosomes (magenta) traveling in a circular path (green arrow) around mitochondria (cyan) on the distal side of the node of Ranvier – Linked to Figure 4B Frame interval: 1.64 s, playback rate = 8 fps, acquisition time = 3 min 11 s, scale bar = 10 μm.

Following this observation, we then used semi-automated tracking of H_C_T-positive signaling endosomes in TA- and soleus-innervating motor axons of the Mito.CFP mouse using the TrackMate plugin^43^ to assess transport dynamics. Strikingly, the mean velocities of retrogradely moving H_C_T-containing signaling endosomes are reduced when approaching the nodal constriction, with a concomitant increase in the relative frequency of pausing. This was followed by an increase in retrograde velocities on the proximal side of the NoR in both TA- (Figure 5A) and soleus-innervating motor axons (Figure S3). Quantitative analyses in TA-innervating axons indicated that the mean velocities of signaling endosomes in the nodal constriction were ∼48% and ∼40% slower compared to the proximal and distal internodal regions, respectively (Figure 5B). Furthermore, the retrograde transport speeds through the proximal internode were ∼14% faster compared to distal internode regions (Figure 5B). In addition, pausing in TA-innervating motor axons was reduced by ∼86% and ∼67% in the proximal and distal internodal regions, respectively, compared to the nodal constriction (Figure 5C). Similarly in soleus-innervating motor axons, the mean velocities of H_C_T-containing signaling endosomes in the nodal constriction were ∼46% and ∼35% slower compared to the proximal and distal internodal regions, respectively (Figures S3B and S3C). As the transport dynamics of signaling endosomes at the NoR largely overlap in FMN and SMN motor axons (Figures 5D, 5E, S3 D, and S3E), we conclude that motor neuron subtypes do not determine overt changes in axonal transport dynamics at the NoR, which is consistent with our previous observations across the larger internodal axonal region^7^

**Figure 6:**
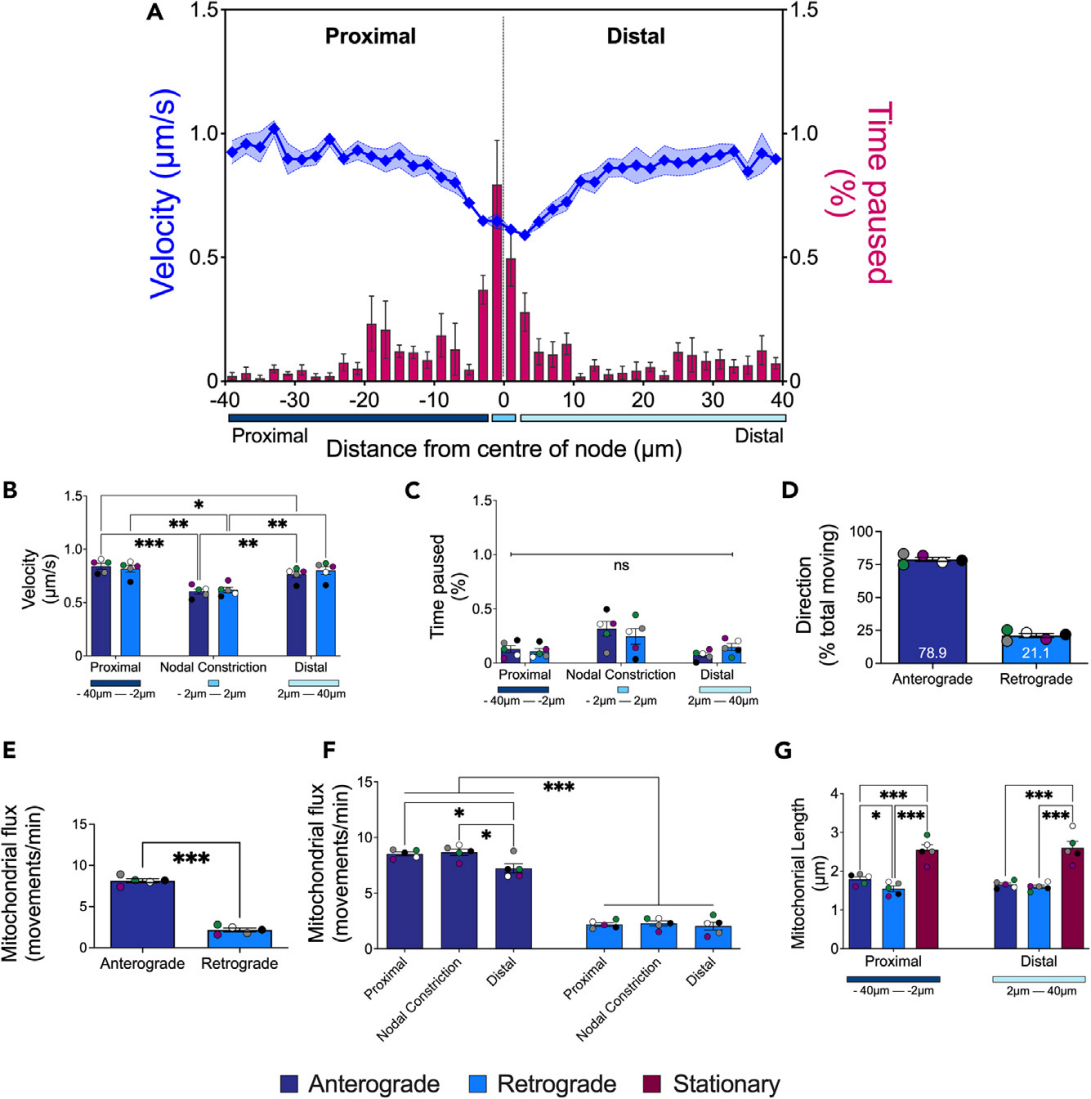
Mitochondrial transport dynamics are bi-directionally altered at the node of Ranvier (A) Combined anterograde and retrograde mitochondrial dynamics (mean moving velocity [blue diamonds] and relative frequency of mean pausing [pink bars]) across the nodal constriction and beyond (80 μm distance) in sciatic nerve axons innervating the tibialis anterior muscle. The x axis represents the distance from the center of the node of Ranvier (μm) and is split into three segments: (1) *Proximal* = 38 μm of the proximal internode (navy); (2) *Center* = 4 μm representing the mean nodal length (as determined in Figure 1D; cyan); and (3) *Distal* = 38 μm of the distal internode (light blue). Comparisons between the nodal constriction, and proximal and distal internodes of *in vivo* axonal transport of anterogradely and retrogradely moving mitochondria of (B) mean moving velocity (∗∗∗Axonal Location: *p* < 0.001; Directionality: *p* = 0.83; Interaction: *p* = 0.19) and (C) relative frequency of mean time paused (∗Axonal Location: *p* = 0.0156; Directionality: *p* = 0.81; Interaction: *p* = 0.39). (D) A higher percentage of mitochondria move in the anterograde than retrograde direction, (E) resulting in greater flux of anterogradely moving mitochondria across all axonal sub-domains (*p* < 0.001). In addition, (F) there is a selective decline in anterograde flux in the distal internode, whereas there is no difference in retrograde flux across axonal sub-domains (Axonal Location: *p* = 0.0021; Directionality: *p* < 0.001; Interaction: *p* = 0.0124). (G) Stationary mitochondria are longer compared to both the anterogradely and retrogradely moving mitochondria, independent of the axonal sub-domain (Axonal Location: *p* = 0.79; Movement: *p* < 0.001; Interaction: *p* = 0.36). For all graphs, data are represented as mean (solid line) ± SEM (dashed lines/error bars). Data were compared by two-way ANOVA and Šídák’s multiple comparisons tests (B, C, F, and G) or an unpaired, two-tailed *t*-test (E). *n* = 5 Mito.CFP mice. ∗*p* < 0.05, ∗∗*p* < 0.01, ∗∗∗*p* < 0.001. See also Figures S4–S6.

To determine whether these phenotypes are specific for the organelle and/or direction of travel, we assessed the axonal transport of mitochondria in TA-innervating motor axons, using the same videos from which signaling endosomes were analyzed (e.g., Figure 5). We separately assessed the anterograde and retrograde axonal transport of mitochondria through the nodal constrictions using the manual tracking feature of TrackMate^43^ (Video S7). Similar to H_C_T-containing signaling endosomes, the mean velocity of mitochondria is also reduced at the nodal constriction, which was then followed by a return to faster velocities for both anterogradely and retrogradely moving mitochondria (Figures 6A and S4). Anterograde mitochondrial velocities in the nodal constriction were ∼28% and ∼21% slower compared to the proximal and distal internodes, respectively (Figure 6B). Similarly, the mean velocities of retrogradely moving mitochondria were ∼24% and ∼23% slower in the nodal constriction compared to the proximal and distal internodal regions, respectively (Figure 6B). We also observed faster mean velocity for anterogradely moving mitochondria in the proximal internode compared to the distal internode (Figure 6B), replicating the increased speeds of signaling endosomes proximal to the node (Figure 5B). However, an increase in pausing frequency is observed only in mitochondria moving anterogradely, but not retrogradely (Figures S4 and 6C). Despite such regional differences in mitochondrial transport, no significant correlation emerged between transport speeds or pausing events and the axonal diameter across each of the individual axonal sub-domains (Figure S5). A higher percentage of mitochondria move anterogradely than retrogradely (Figure 6D), with mitochondrial flux being more than three times greater in the anterograde than retrograde direction (Figure 6E). Consistent with the accumulation distal to the node, anterograde mitochondrial flux is lower in the distal internode than the preceding axonal subdomains, whereas no regional differences in retrograde flux were found (Figure 6F). This suggests that anterogradely moving mitochondria are the main source for nodal accumulation. Finally, we determined that independently from their location, stationary mitochondria are longer than motile mitochondria, and that, only in the proximal internode, retrogradely moving mitochondria are shorter than those moving anterogradely (Figures 6G and S6).

Video S7. Representative videos of tracked mitochondria separated by direction of travel – Linked to Figure 6 and Figure S4 (A) Representative unprocessed time-lapse video. Using the TrackMate plugin in FIJI/ImageJ, mitochondria (green dots and lines) were manually tracked in the (B) anterograde (magenta) and (C) retrograde (gold) directions. Frame interval: 1.77 s, playback rate = 20 fps, acquisition time = 10 min 21 s, scale bar: 10 μm.

In summary, we have characterized an *in vivo* axonal transport profile of signaling endosomes and mitochondria across the NoR in multiple motor neuron sub-types within the sciatic nerve.

## Discussion

Axons are reliant upon efficient transport to maintain neuronal homeostasis. Using our intravital imaging approach,^38^^,^^44^ we aimed to resolve the *in vivo* axonal transport dynamics of multiple organelles at the NoR in intact and synaptically connected motor axons in the sciatic nerves of healthy mice. Firstly, we observed equivalent morphological features of the NoR in motor axons innervating prototypical fast and slow muscles. Next, we identified the co-clustering of signaling endosomes and mitochondria specifically at the distal side of the NoR. *In vivo* axonal transport analyses revealed a slowing of mitochondria and signaling endosomes as they approach the NoR, with a concomitant rise in pausing events. This was followed by an increase in velocity in the adjacent intranodal region, irrespective of directionality and motor neuron subtype. Collectively, these findings further our understanding of the morphology and physiology of NoR in peripheral nerve axons.

### Node of Ranvier morphology in peripheral nerve

We have previously reported that peripheral motor axons transporting H_C_T upon uptake at neuromuscular junctions are larger in diameter than H_C_T-positive peripheral sensory axons innervating the same muscle,^9^ consistent with the observation that ventral roots have a larger average caliber than dorsal roots in healthy mice.^45^ Such differences have also been shown in central nervous system (CNS) axons^46^ and in ventral funiculi axons in the thoracic spinal cord.^47^ Conversely, the mean intranodal diameters of motor axons innervating the TA, lateral gastrocnemius, and soleus muscles do not differ.^7^

Here, we reveal comparable morphological features of the NoR between H_C_T-labeled FMN and SMN axons, as well as between ChAT.eGFP and Mito.CFP FMN axons, which are in turn, also comparable to the previously reported sciatic nerve profiles^47^ and morphometric results obtained from tibial nerve explants.^25^ In contrast, morphological features of the NoR in CNS axons, such as those in the rat optic nerve and cerebral cortex, are heterogeneous, with differences observed between axons rather than within axons, even when comparing axons with similar intranodal diameters.^30^ However, altering neuronal activity can modify NoR properties,^48^ suggesting the axonal morphology is dynamically regulated.

### Organelle accumulations at the NoR

Using intravital imaging, we show dual-organelle accumulation at the NoR *in vivo*. As the peak fluorescence signal from both signaling endosomes and mitochondria was approximately 3–4.5 μm distal to the center of the NoR, it is likely that these clusters were located in the distal juxtaparanode region. Indeed, the peak signal from the distal region was approximately 50% greater than from the proximal internode for both organelles. Our data are in line with previous reports of expansions in the distal juxtaparanode of myelinated axons, which have been attributed to accumulations of diverse organelles, including retrogradely transported glycoproteins,^28^ retrogradely transported horseradish peroxidase (HRP),^27^ multivesicular bodies,^18^ lysosomes^29^ and mitochondria.^21^ However, there may be regional and/or neuronal subtype differences in organelle clustering at the NoR, since mitochondria do not accumulate at the NoR in small-diameter myelinated CNS axons,^49^ nor in the optic nerve.^50^

Mechanistically, it is unclear whether organelle accumulation at the NoR is an active process, or is simply a consequence of a localized cytoskeletal bottleneck. Although neurofilaments are reduced up to 10-fold at the NoR,^25^^,^^26^^,^^51^ the microtubule network must be preserved to enable axonal transport over the long distances covered by peripheral nerve axons.^52^ Hence, the accumulation of organelles at the NoR might be structurally and functionally linked, with several potential mechanisms that could cause such focal accumulations, including differences in: 1) axonal protein translation^53^; 2) metabolic demands^23^; 3) axo-glial communication^54^; 4) activity dependent alterations to microtubule structure^55^; 5) differential distributions of motor proteins and their regulators^12^; 6) hotspots for cytoskeletal anchoring (e.g., syntaphilin-mediated)^56^; or 7) microtubule post-translational modifications.^57^ Furthermore, myelination and axonal electrical activity also play a regulatory role in mitochondrial localization at the CNS NoR.^58^

On the other hand, arrested mitochondrial motility is linked to activity-dependent axonal Ca^2+^ elevations.^22^^,^^59^ Furthermore, radial contractility alters axonal size to accommodate the movements of larger organelles.^60^ Thus, focal organelle accumulations might be linked to activity and can be transient in nature; however considerable follow up assessments are required to determine their mechanisms and dynamics. Moreover, direct interactions between Rab7-positive signaling endosomes and mitochondria, as well as co-transport with other organelles, could also influence nodal accumulations.^61^^,^^62^^,^^63^

### Organelle axonal transport dynamics at the nodes of Ranvier

While we have previously characterized signaling endosome and mitochondrial transport in physiological and pathological conditions,^7^^,^^9^^,^^64^^,^^65^ this study quantitatively assesses *in vivo* axonal transport dynamics of these organelles specifically at the NoR. We observed similar patterns of transport dynamics of signaling endosomes and mitochondria across the node, with both organelles displaying faster velocities at the internodes, and more pausing at the NoR, which contrasts with the report of transient accelerations of neurofilaments through nodal constrictions.^25^ Such transport differences between signaling endosomes and mitochondria compared to neurofilaments might be directly attributed to the transport machinery that regulates fast and slow axonal transport or their unique biological functions.^66^^,^^67^ We also identified faster speeds in the proximal internode compared to the distal internode of both retrogradely transported signaling endosomes and anterogradely, but not retrogradely, moving mitochondria. This, along with the observations of organelle accumulations at the distal NoR, is suggestive of asymmetrical structural or functional differences in the nodal compartments of peripheral motor axons, which warrants further investigation. Given the similar composition and function of the NoR and the axon initial segment (e.g., distribution of Na_V_1.6 channels,^68^ Ank-G,^69^ and Neurofascin-186^70^), it would be interesting to compare transport dynamics between these two key axonal specializations.

Mitochondrial axonal transport ensures that specific neuronal regions, such as synapses and NoRs, can respond to localized high energy and Ca^2+^ handling demands.^71^^,^^72^^,^^73^ We speculate that the observed mitochondrial axonal transport phenotypes are linked to the Ca^2+^ dynamics at the NoR. Indeed, an increase in intracellular Ca^2+^ decreases mitochondrial motility, independent of directionality, with Miro directly interacting with the motor domain of kinesin-1 to prevent its binding to microtubules.^22^^,^^74^ Electrical activity also regulates mitochondrial dynamics, as Na^+^ channel activation in the nodal and paranodal regions slows mitochondrial transport (coupled to Na/K-ATPase),^22^ and can even modulate the size of stationary mitochondria by influencing fission/fusion events.^58^ However, we did not observe spatial differences in the lengths of stationary mitochondria, but rather we detected major differences in mitochondrial length between stationary and motile mitochondria. On the other hand, kinases, such as GSK3, JNK3, and p38 MAPK, regulate kinesin-1 and/or cytoplasmic dynein motor protein activity and are considered as key modifiers in neurodegeneration.^2^^,^^10^ Indeed, we and others have previously reported that inhibiting p38 MAPK rescues axonal transport deficits in a variety of models of motor neuron disease (MND).^75^^,^^76^^,^^77^

The physiological role of signaling endosome trafficking at the NoR is inherently different from that of mitochondria. In motor neurons, signaling endosomes are Rab7-positive organelles that deliver distally activated signaling complexes from axon termini to the cell body to impact transcription and translation across the neuron.^78^ In addition to the factors mentioned above (e.g., Ca^2+^, electrical activity, and kinases), the trafficking journey of signaling endosomes can be modulated by additional factors, including neurotrophins and their receptors^7^^,^^31^^,^^35^^,^^79^^,^^80^^,^^81^^,^^82^^,^^83^ and components of the extracellular matrix,^34^ with glycolytic enzymes (e.g., GAPDH) sufficient to provide the in-transit ATP necessary for processive movement.^84^^,^^85^ The reduction in speed and increased pausing that we observed at the NoR might be caused by the cytoskeletal constraints associated with the reduced axonal diameter. On the other hand, Rab7-positive organelles are involved in local mRNA translation^61^ and the majority of axonal mRNAs are undergoing active translation,^86^ which may link the signaling endosome transport and accumulation phenotypes with local translation at the NoR.^87^ Additionally, the accumulation of synaptic vesicle proteins, including SV2 and synaptophysin, at the NoR has been reported to function in axonal membrane processing and/or turnover.^88^ Determining the precise molecular mechanisms regulating axonal transport dynamics at the NoR and their physiological roles will be important targets of future experiments.

In conclusion, using intravital imaging, we have characterized NoR morphologies in FMNs and SMNs, identified accumulations of signaling endosomes and mitochondria at the distal NoR, and determined the axonal transport dynamics of both organelles through the NoR. Finally, this work has clear implications for the peripheral nervous system and its numerous disorders (e.g., MND, multiple sclerosis),^89^^,^^90^ and suggests that both the internodal and nodal transport modalities should be monitored across neurodegenerative disease models.

### Limitations of the study

Our axonal transport method enables the *in vivo* visualization of both signaling endosomes and mitochondria from the same axon. Due to several factors, we performed manual tracking of the organelles, which might lead to subjective differences between the researchers performing the tracking. To minimize this risk, the same individual tracked the entire dataset (i.e., FA for signaling endosomes; APT for mitochondria). Furthermore, to avoid potential bias, quantitative analyses of the tracking outputs were only analyzed upon the completion of organelle tracking from all videos. Tracking was performed by randomly selecting a minimum of 20 endosomes/mitochondria per axon. We assume that the parameters gleaned from this analysis (e.g., mean speed, pausing events, and so forth) carried out in three or more axons per animal, accurately represent the physiological state of the organelles, the axons, and the individual mice. Another limitation of our study is that we assume that organelle dynamics at the NoR are similar between peripheral nerves. To address this point experimentally, validation of our conclusions in forelimb^8^/cranial nerves should be carried out in future studies.

## Resource availability

### Lead contact

Further information and requests for resources and reagents should be directed to and will be fulfilled by the Lead Contact, Giampietro Schiavo (giampietro.schiavo@ucl.ac.uk).

### Materials availability

Requests for resources used in this study are available from the lead contact.

### Data and code availability

• **Data:** All data reported in this article will be shared by the lead contact upon request.
• **Code:** This article does not report any original code.
• **All other items:** Any additional information required to reanalyze the data reported in this article is available from the lead contact upon request.

## Acknowledgments

We thank the personnel of the Denny Brown Laboratories (Queen Square Institute of Neurology, 10.13039/501100000765University College London) for assistance in maintaining the mouse colonies, and Elena R. Rhymes and David Villarroel-Campos (Queen Square Institute of Neurology, 10.13039/501100000765University College London) for critical reading of the article. This work was supported by a Junior Non-Clinical Fellowship from the 10.13039/501100000406Motor Neurone Disease Association (Tosolini/Oct20/973-799) (APT); a Col Bambrick MND Research Grant from Motor Neuron Disease Research Australia (IG 2450) (APT); a FightMND Drug Development Grant awarded to Giovanni Nardo (10.13039/100015974Istituto di Ricerche Farmacologiche Mario Negri - IRCCS) (DDG-73; for APT); EMBO short-term fellowship (SN); Medical Research Council fellowships (MR/S006990/1 and MR/Y010949/1) (JNS); Wellcome Trust Senior Investigator Awards (107116/Z/15/Z and 223022/Z/21/Z) (GS), and a UK Dementia Research Institute award (UKDRI-1005) (GS).

## Author contributions

Conceptualization: APT and GS; methodology: APT, FA, SN, and JNS; Investigation: APT, FA, and SN; writing – original draft: APT; writing – review and editing: APT, SN, JNS and GS; funding acquisition: APT and GS; resources: APT, SN, JNS, and GS; supervision: APT and GS.

## Declaration of interests

The authors declare no competing interests.

## STAR★Methods

### Key resources table

**Table:** 

REAGENT or RESOURCE	SOURCE	IDENTIFIER
**Antibodies**		

Na_V_1.6 [SCN8a]	Alomone Labs	Cat # ASC-009 RRID AB_2040202
Anti-CASPR/Neurexin IV	Antibodies Incorporated	Cat# 75-001 RRID:AB_2083496
Goat anti-Mouse IgG (H + L) Cross-Adsorbed Secondary Antibody, Alexa Fluor™ 555	Thermo Fisher Scientific	Cat# A-21422 RRID:AB_2535844
Donkey anti-Rabbit IgG (H + L) Highly Cross-Adsorbed Secondary Antibody, Alexa Fluor™ 647	Thermo Fisher Scientific	Cat# A-31573 RRID:AB_2536183
Alexa Fluor 555 C2 maleimide	Thermo Fisher Scientific	Cat# A-20346

**Chemicals, peptides, and recombinant proteins**		

H_C_T 441	Restani et al.^84^	N/A

**Experimental models: organisms/strains**		

Mouse: Tg(Chat-EGFP)GH293Gsat/Mmucd (ChAT-eGFP)	Mutant Mouse Resource and Research Center	RRID: MMRRC: 000296-UCD
Mouse: B6.Cg-Tg(Thy1-CFP/COX8A)S2Lich/J (Mito.CFP)	Jackson Laboratory	RRID: IMSR_JAX: 007967
Mouse: B6; CBA-Tg(Plp1-EGFP)10Wmac/J (PLP-GFP)	Jackson Laboratory	RRID: IMSR_JAX:033357

**Software and algorithms**		

TrackMate plugin	FIJI/ImageJ	Ershov et al.^41^
Prism 10 (Version 10.2.3)	GraphPad	https://www.graphpad.com/features

**Other**		

Fluoromount G Mounting Medium	Thermo Fisher Scientific	Cat# 00-4958-02 RRID: SCR_015961
Dako fluorescence mounting medium	Agilent Technologies	Cat# S3023

### Experimental model and study participant details

#### Animals

Animal experiments performed in the United Kingdom were conducted in accordance with the European Community Council Directive of 24 November 1986 (86/609/EEC), under license from the UK Home Office in accordance with the Animals (Scientific Procedures) Act 1986, and were approved by the UCL Institute of Neurology Ethical Review Committee. Procedures carried out in Italy were approved by the ethical committee and by the animal welfare coordinator of the OPBA from the University of Padua. All procedures specified in the projects are approved by the Italian Ministry of Health, Ufficio VI (authorisation numbers: 359/2015PR; 81/2017 PR; 521/2018 PR; 439/2019 PR) and were conducted in accordance with National laws and policies (D.L. n. 26, March 14, 2014), following the guidelines established by the European Community Council Directive (2010/63/EU) for the care and use of animals for scientific purposes.

3-6 month old heterozygous male and female mice of the following transgenic strains were used: 1) Tg(Chat-EGFP)GH293Gsat/Mmucd mice (RRID: MMRRC_000296-UCD,^91^; referred to as ‘ChAT.eGFP’ mice; 2) B6.Cg-Tg(Thy1-CFP/COX8A)S2Lich/J (RRID: IMSR_JAX: 007967),^92^ referred to as ‘Mito.CFP’ mice; and 3) B6; CBA-Tg(Plp1-EGFP)10Wmac/J (RRID: IMSR_JAX:033357),^93^ referred to as ‘PLP-GFP’ mice. As we have previously determined that sex does not influence basal transport dynamics in ChAT.eGFP mice,^9^ all results are from both males and females. Mice were housed in individually ventilated cages in a controlled temperature/humidity environment and maintained on a 12 h light/dark cycle with access to food and water *ad libitum*.

### Method details

#### Intramuscular injections of HcT

Fluorescently labeled atoxic fragment of tetanus neurotoxin (H_C_T-555) was prepared as previously described.^94^ Briefly, H_C_T (residues 875–1315) fused to an improved cysteine-rich region was expressed in bacteria as a glutathione-S-transferase fusion protein, cleaved and subsequently labeled with Alexa Fluor 555 C2 maleimide (Thermo Fisher Scientific, A-20346). Mice were anesthetized using isoflurane, and after the fur on the ventrolateral lower leg was shaved, mice were placed on a heat-pad ready for intramuscular injections. A small incision was made on the ventral surface below the patella for the tibialis anterior muscle (TA), whereas for the soleus muscle a vertical incision was made on the skin covering the lateral surface of lower hindlimb between the patella and tarsus to expose the underlying musculature. Guided by previously established motor endplate maps,^95^ intramuscular injections were performed, with 7.5–10 μg of H_C_T in PBS in a volume of ∼3.5 μL using a 701 N Hamilton syringe (Merck, 20,779) for TA, or 1 μL in PBS using pulled graduated, glass micropipettes (Drummond Scientific,5-000-1001-X10) for soleus, as previously described.^96^ Upon H_C_T administration, the skin was sutured, and mice were monitored for up to 1 h before returning to the home cage.

#### *In vivo* imaging

Signaling endosomes were visualised *in vivo* after administration of H_C_T, as previously described.^38^^,^^44^^,^^97^ At least 4 h after HcT intramuscular injections, mice were re-anaesthetised with isoflurane, and the sciatic nerve was exposed by first removing the skin and then the overlying biceps femoris muscle. To aid the imaging process a small piece of parafilm was inserted between the underlying connective tissue and the sciatic nerve. The anesthetized mouse and nosepiece were then transferred to an inverted LSM780 confocal microscope (Zeiss) enclosed within an environmental chamber maintained at 37°C. Superficially located axons containing H_C_T-loaded signaling endosomes were selected at random for imaging, as previously described.^38^^,^^44^^,^^97^Time-lapse microscopy was performed using a 40×, 1.3 NA DIC Plan-Apochromat oil-immersion objective (Zeiss) focusing on axons around the NoR using an 80× digital zoom (1024 × 1024, <1% laser power). Frame intervals of ∼0.4–0.5 s were used when acquiring transport videos of motile H_C_T-positive signaling endosomes, whereas frame intervals of ∼1.5–2.0 s were used when acquiring videos of motile Mito.CFP-positive mitochondria alone or in combination with H_C_T-positive signaling endosomes. All imaging was concluded within a maximum of 1 h from initiating re-anaesthesia.

#### Morphological analysis of the NoR

Axonal morphologies at the NoR were assessed using the H_C_T transport videos obtained in TA- or soleus-innervating motor axons from ChAT.eGFP and Mito.CFP mice, as previously described.^7^^,^^9^ Orthogonal measurements were made between the upper and lower or proximal and distal ends of axonal regions containing motile H_C_T signaling endosomes (Figure 1A). A minimum of ten measurements (i.e., combined proximal and distal internodal axonal diameter, axon diameter in the nodal constriction, and axon length in the nodal constriction) from at least three different axons were used to calculate the average diameter/length per animal.

#### Spatial analysis of organelles at the NoR

The distributions of signaling endosomes and mitochondria at the NoR were assessed from the axonal H_C_T transport videos in TA- or soleus-innervating motor axons in Mito.CFP mice. Using FIJI/ImageJ, the relative fluorescence profiles of H_C_T-containing signaling endosomes and mitochondria across the NoR and internodal regions were measured after drawing an 80 μm region centered around the nodal constriction from the z stack projection. After the background was subtracted, the relative fluorescence intensity profiles were then plotted, and averaged from a minimum of 3 axons per animal.

#### Immunohistochemistry of the sciatic nerve

##### NoR staining

Sciatic nerves were dissected from euthanised ChAT.eGFP mice and fixed in 4% paraformaldehyde in PBS for approximately 1 h at room temperature. Sciatic nerves were washed in PBS three times for 5 min, teased into individual fibers/bundles and were then permeabilised and blocked in a blocking solution containing 1% Triton X-100 and 10% bovine serum albumin in PBS for 1 h at room temperature. Sciatic nerve fibers were then incubated in a blocking solution containing primary antibodies against Na_V_1.6 [SCN8a] (1:400; Alomone, ASC-009) and Caspr [clone K65/35] (1:500; Antibodies Incorporated 75-001) for ∼3 days at 4°C with mild agitation. Following three washes in PBS at room temperature, fibers were then immersed in a solution containing anti-mouse-555 (1:500; Thermo Fisher Scientific; A-21422) and anti-rabbit-647 (1:500; Thermo Fisher Scientific; A31573) secondary antibodies in PBS for ∼2 h at room temperature. Fibers were then washed three times in PBS, mounted in Fluoromount-G (Thermo Fisher Scientific, 00-4958-02) and covered with 22 × 50 mm cover glass (VWR, 631-0137). Slides were dried and imaged with a LSM780 confocal microscope using a 63× Plan-Apochromat oil immersion objective (Zeiss).

##### H_C_T and Schwann cell cytosol

Anesthetized PLP-GFP mice were injected into the TA muscle with H_C_T^38^; after 24 h, mice were euthanised and the sciatic nerves dissected and fixed in 4% paraformaldehyde in PBS for 2 h at room temperature. After 3 × 5 min washes in PBS, sciatic nerves were then de-sheathed, teased into individual fibers and mounted using Dako fluorescence mounting medium (Agilent Technologies, cat S3023). z stack images were obtained using a with a confocal microscope (Zeiss LSM900 Airyscan2) equipped with an EC Plan-Neofluar 40×/1.30 oil objective.

#### Tracking analysis

Confocal “.czi” images were opened in FIJI/ImageJ (http://rsb.info.nih.gov/ij/), converted to “.tiff”, and using the TrackMate plugin^43^ were tracked in a semi-automatic way for H_C_T-positive signaling endosomes^7^ or manually for mitochondria.^98^ The following criteria were used for the tracking analysis: 1) only endosomes and mitochondria that were moving for ≥10 consecutive frames were included, and terminal pauses, as defined by the absence of movement in ≥10 consecutive frames, were excluded; 2) each axon required a minimum of 20 trackable organelles per axon, except for retrogradely moving mitochondria, which only required a minimum of 10 per axon; 3) at least three separate axons were assessed per mouse. A pause was defined by a previously motile organelle with a velocity of ≤0.1 μm/s between consecutive frames (to account for a potential breathing/arterial pulsing artifact), and the time paused (%) was determined by dividing the number of pauses by the total number of frame-to-frame movements assessed from an individual axon.

Transport dynamics of the retrogradely moving H_C_T signaling endosomes, as well as anterogradely and retrogradely moving mitochondria were separately assessed with TrackMate,^43^ using the above criteria. Each frame-to-frame recording (e.g., velocity, pausing) was matched with its x and y co-ordinates, binned every 2 μm across an 80 μm axonal length centered at the NoR, and subsequently separated into either the proximal internode, nodal constriction, or distal internode locations. The mean moving velocity represents the average of all frame-to-frame movements in a particular axonal area (excluding pausing events), whereas the relative frequency of mean pausing was determined by assessing the relative number of pauses in relation to the axonal location. The correlation figures were generated by plotting the mean velocity of signaling endosome and mitochondrion transport, as well as the relative frequency of signaling endosome and mitochondrion pausing, against the mean value of axonal diameters for each individual axon.

#### Mitochondrial directionality, flux and length

Mitochondrial directionality, flux and length were quantified using the same videos used to analyze *in vivo* axonal mitochondrial transport at the NoR. For directionality, every movement was separately counted for individual anterogradely and retrogradely moving mitochondria across the proximal, nodal constriction and distal regions using Cell Counter plugin (FIJI). Outputs were averaged across the subdomains, and then presented as a relative fraction directly comparing anterogradely with retrogradely moving mitochondria from individual axons, and then averaged across the three axons per animal. For mitochondrial flux, the outputs from the directionality assessments were then normalised to the time of individual videos and presented as the mean number of moving mitochondria per minute, either combining the axonal subdomains (e.g., Figure 6E) or presenting them separately for both anterogradely and retrogradely moving mitochondria (e.g., Figure 6F). For mitochondrial length, a minimum of 10 randomly selected stationary, anterogradely or retrogradely moving mitochondria from the proximal and distal regions were measured from every axonal transport video, and presented for individual mice as the mean value from a minimum of 3 axons (Figure 6F) or individually (Figure S6).

### Quantification and statistical analysis

GraphPad Prism 10 Software (Version 10.2.3) was used for all statistical analyses and figure production. Data were assumed to be normally distributed, and parametric data were assessed using paired or unpaired two-tailed *t*-tests, as well as one-way or two-way analyses of variance (ANOVA) with Holm-Sidaks multiple comparison tests. Pearson correlation coefficients (two-tailed) were also computed. Specific statistical details of each experiment can be found in the figure legends. n = number of animals. ∗*p* < 0.05, ∗∗*p* < 0.01, ∗∗∗*p* < 0.001.

## References

[1] Maday S., Twelvetrees A.E., Moughamian A.J., Holzbaur E.L.F. (2014). Axonal Transport: Cargo-Specific Mechanisms of Motility and Regulation. Neuron.

[2] Brady S.T., Morfini G.A. (2017). Regulation of motor proteins, axonal transport deficits and adult-onset neurodegenerative diseases. Neurobiol. Dis..

[3] Sleigh J.N., Rossor A.M., Fellows A.D., Tosolini A.P., Schiavo G. (2019). Axonal transport and neurological disease. Nat. Rev. Neurol..

[4] Stifani N. (2014). Motor neurons and the generation of spinal motor neuron diversity. Front. Cell. Neurosci..

[5] Blum J.A., Klemm S., Shadrach J.L., Guttenplan K.A., Nakayama L., Kathiria A., Hoang P.T., Gautier O., Kaltschmidt J.A., Greenleaf W.J., Gitler A.D. (2021). Single-cell transcriptomic analysis of the adult mouse spinal cord reveals molecular diversity of autonomic and skeletal motor neurons. Nat. Neurosci..

[6] Ragagnin A.M.G., Shadfar S., Vidal M., Jamali M.S., Atkin J.D. (2019). Motor Neuron Susceptibility in ALS/FTD. Front. Neurosci..

[7] Tosolini A.P., Sleigh J.N., Surana S., Rhymes E.R., Cahalan S.D., Schiavo G. (2022). BDNF-dependent modulation of axonal transport is selectively impaired in ALS. Acta Neuropathol. Commun..

[8] Lang Q., Schiavo G., Sleigh J.N. (2023). In vivo imaging of axonal transport in peripheral nerves of rodent forelimbs. Neuronal Signal..

[9] Sleigh J.N., Tosolini A.P., Gordon D., Devoy A., Fratta P., Fisher E.M.C., Talbot K., Schiavo G. (2020). Mice Carrying ALS Mutant TDP-43, but Not Mutant FUS, Display In Vivo Defects in Axonal Transport of Signaling Endosomes. Cell Rep..

[10] Gibbs K.L., Greensmith L., Schiavo G. (2015). Regulation of Axonal Transport by Protein Kinases. Trends Biochem. Sci..

[11] Nirschl J.J., Ghiretti A.E., Holzbaur E.L.F. (2017). The impact of cytoskeletal organization on the local regulation of neuronal transport. Nat. Rev. Neurosci..

[12] Cason S.E., Holzbaur E.L.F. (2022). Selective motor activation in organelle transport along axons. Nat. Rev. Mol. Cell Biol..

[13] Berth S.H., Lloyd T.E. (2023). Disruption of axonal transport in neurodegeneration. J. Clin. Invest..

[14] D’Este E., Kamin D., Balzarotti F., Hell S.W. (2017). Ultrastructural anatomy of nodes of Ranvier in the peripheral nervous system as revealed by STED microscopy. Proc. Natl. Acad. Sci..

[15] Rasband M.N., Peles E. (2021). Mechanisms of node of Ranvier assembly. Nat. Rev. Neurosci..

[16] Johnson C., Holmes W.R., Brown A., Jung P. (2015). Minimizing the caliber of myelinated axons by means of nodal constrictions. J. Neurophysiol..

[17] Reles A., Friede R.L. (1991). Axonal cytoskeleton at the nodes of Ranvier. J. Neurocytol..

[18] Berthold C.-H., Fabricius C., Rydmark M., Andersén B. (1993). Axoplasmic organelles at nodes of Ranvier. I. Occurrence and distribution in large myelinated spinal root axons of the adult cat. J. Neurocytol..

[19] Hsieh S.T., Kidd G.J., Crawford T.O., Xu Z., Lin W.M., Trapp B.D., Cleveland D.W., Griffin J.W. (1994). Regional modulation of neurofilament organization by myelination in normal axons. J. Neurosci..

[20] Tsukita S., Ishikawa H. (1981). The cytoskeleton in myelinated axons: serial section study. Biomed. Res..

[21] Fabricius C., Berthold C.-H., Rydmark M. (1993). Axoplasmic organelles at nodes of Ranvier. II. Occurrence and distribution in large myelinated spinal cord axons of the adult cat. J. Neurocytol..

[22] Zhang C.L., Ho P.L., Kintner D.B., Sun D., Chiu S.Y. (2010). Activity-Dependent Regulation of Mitochondrial Motility by Calcium and Na/K-ATPase at Nodes of Ranvier of Myelinated Nerves. J. Neurosci..

[23] Chiu S.Y. (2011). Matching Mitochondria to Metabolic Needs at Nodes of Ranvier. Neuroscientist.

[24] Brown A. (2003). Axonal transport of membranous and nonmembranous cargoes. J. Cell Biol..

[25] Walker C.L., Uchida A., Li Y., Trivedi N., Fenn J.D., Monsma P.C., Lariviére R.C., Julien J.-P., Jung P., Brown A. (2019). Local Acceleration of Neurofilament Transport at Nodes of Ranvier. J. Neurosci..

[26] Ciocanel M.-V., Jung P., Brown A. (2020). A mechanism for neurofilament transport acceleration through nodes of Ranvier. Mol. Biol. Cell.

[27] Berthold C.-H., Mellström A. (1982). Distribution of peroxidase activity at nodes of Ranvier after intramuscular administration of horseradish peroxidase in the cat. Neuroscience.

[28] Armstrong R., Toews A.D., Morell P. (1987). Axonal transport through nodes of Ranvier. Brain Res..

[29] Gatzinsky K.P., Berthold C.-H. (1990). Lysosomal activity at nodes of Ranvier during retrograde axonal transport of horseradish peroxidase in alpha-motor neurons of the cat. J. Neurocytol..

[30] Arancibia-Cárcamo I.L., Ford M.C., Cossell L., Ishida K., Tohyama K., Attwell D. (2017). Node of Ranvier length as a potential regulator of myelinated axon conduction speed. Elife.

[31] Sleigh J.N., Villarroel-Campos D., Surana S., Wickenden T., Tong Y., Simkin R.L., Vargas J.N.S., Rhymes E.R., Tosolini A.P., West S.J. (2023). Boosting peripheral BDNF rescues impaired in vivo axonal transport in CMT2D mice. JCI Insight.

[32] Delezie J., Weihrauch M., Maier G., Tejero R., Ham D.J., Gill J.F., Karrer-Cardel B., Rüegg M.A., Tabares L., Handschin C. (2019). BDNF is a mediator of glycolytic fiber-type specification in mouse skeletal muscle. Proc. Natl. Acad. Sci. USA.

[33] Bercsenyi K., Schmieg N., Bryson J.B., Wallace M., Caccin P., Golding M., Zanotti G., Greensmith L., Nischt R., Schiavo G. (2014). Tetanus toxin entry. Nidogens are therapeutic targets for the prevention of tetanus. Science.

[34] Surana S., Villarroel-Campos D., Rhymes E.R., Kalyukina M., Panzi C., Novoselov S.S., Fabris F., Richter S., Pirazzini M., Zanotti G. (2024). The tyrosine phosphatases LAR and PTPRδ act as receptors of the nidogen-tetanus toxin complex. EMBO J..

[35] Deinhardt K., Salinas S., Verastegui C., Watson R., Worth D., Hanrahan S., Bucci C., Schiavo G. (2006). Rab5 and Rab7 Control Endocytic Sorting along the Axonal Retrograde Transport Pathway. Neuron.

[36] Goto-Silva L., McShane M.P., Salinas S., Kalaidzidis Y., Schiavo G., Zerial M. (2019). Retrograde transport of Akt by a neuronal Rab5-APPL1 endosome. Sci. Rep..

[37] Surana S., Tosolini A.P., Meyer I.F., Fellows A.D., Novoselov S.S., Schiavo G. (2018). The travel diaries of tetanus and botulinum neurotoxins. Toxicon.

[38] Tosolini A.P., Villarroel-Campos D., Schiavo G., Sleigh J.N. (2021). Expanding the Toolkit for In Vivo Imaging of Axonal Transport. J. Vis. Exp..

[39] Negro S., Lessi F., Duregotti E., Aretini P., La Ferla M., Franceschi S., Menicagli M., Bergamin E., Radice E., Thelen M. (2017). CXCL12α/SDF-1 from perisynaptic Schwann cells promotes regeneration of injured motor axon terminals. EMBO Mol. Med..

[40] Rodella U., Negro S., Scorzeto M., Bergamin E., Jalink K., Montecucco C., Yuki N., Rigoni M. (2017). Schwann cells are activated by ATP released from neurons in an in vitro cellular model of Miller Fisher syndrome. Dis. Model. Mech..

[41] Negro S., Lauria F., Stazi M., Tebaldi T., D’Este G., Pirazzini M., Megighian A., Lessi F., Mazzanti C.M., Sales G. (2022). Hydrogen peroxide induced by nerve injury promotes axon regeneration via connective tissue growth factor. Acta Neuropathol. Commun..

[42] Wang T., Li W., Martin S., Papadopulos A., Joensuu M., Liu C., Jiang A., Shamsollahi G., Amor R., Lanoue V. (2020). Radial contractility of actomyosin rings facilitates axonal trafficking and structural stability. J. Cell Biol..

[43] Ershov D., Phan M.-S., Pylvänäinen J.W., Rigaud S.U., Le Blanc L., Charles-Orszag A., Conway J.R.W., Laine R.F., Roy N.H., Bonazzi D. (2022). TrackMate 7: integrating state-of-the-art segmentation algorithms into tracking pipelines. Nat. Methods.

[44] Sleigh J.N., Tosolini A.P., Schiavo G. (2020). In Vivo Imaging of Anterograde and Retrograde Axonal Transport in Rodent Peripheral Nerves. Methods Mol. Biol..

[45] Rossor A.M., Sleigh J.N., Groves M., Muntoni F., Reilly M.M., Hoogenraad C.C., Schiavo G. (2020). Loss of BICD2 in muscle drives motor neuron loss in a developmental form of spinal muscular atrophy. Acta Neuropathol. Commun..

[46] Ford M.C., Alexandrova O., Cossell L., Stange-Marten A., Sinclair J., Kopp-Scheinpflug C., Pecka M., Attwell D., Grothe B. (2015). Tuning of Ranvier node and internode properties in myelinated axons to adjust action potential timing. Nat. Commun..

[47] Perrot R., Lonchampt P., Peterson A.C., Eyer J. (2007). Axonal neurofilaments control multiple fiber properties but do not influence structure or spacing of nodes of Ranvier. J. Neurosci..

[48] Cullen C.L., Pepper R.E., Clutterbuck M.T., Pitman K.A., Oorschot V., Auderset L., Tang A.D., Ramm G., Emery B., Rodger J. (2021). Periaxonal and nodal plasticities modulate action potential conduction in the adult mouse brain. Cell Rep..

[49] Edgar J.M., McCulloch M.C., Thomson C.E., Griffiths I.R. (2008). Distribution of mitochondria along small-diameter myelinated central nervous system axons. J. Neurosci. Res..

[50] Perge J.A., Koch K., Miller R., Sterling P., Balasubramanian V. (2009). How the optic nerve allocates space, energy capacity, and information. J. Neurosci..

[51] Rydmark M. (1981). Nodal axon diameter correlates linearly with internodal axon diameter in spinal roots of the cat. Neurosci. Lett..

[52] Hahn I., Voelzmann A., Liew Y.-T., Costa-Gomes B., Prokop A. (2019). The model of local axon homeostasis - explaining the role and regulation of microtubule bundles in axon maintenance and pathology. Neural Dev..

[53] Rangaraju V., Tom Dieck S., Schuman E.M. (2017). Local translation in neuronal compartments: how local is local?. EMBO Rep..

[54] Ronzano R., Roux T., Thetiot M., Aigrot M.S., Richard L., Lejeune F.X., Mazuir E., Vallat J.M., Lubetzki C., Desmazières A. (2021). Microglia-neuron interaction at nodes of Ranvier depends on neuronal activity through potassium release and contributes to remyelination. Nat. Commun..

[55] Peña-Ortega F., Robles-Gómez Á.A., Xolalpa-Cueva L. (2022). Microtubules as Regulators of Neural Network Shape and Function: Focus on Excitability, Plasticity and Memory. Cells.

[56] Kang J.-S., Tian J.-H., Pan P.-Y., Zald P., Li C., Deng C., Sheng Z.-H. (2008). Docking of Axonal Mitochondria by Syntaphilin Controls their Mobility and Affects Short-term Facilitation. Cell.

[57] Janke C., Magiera M.M. (2020). The tubulin code and its role in controlling microtubule properties and functions. Nat. Rev. Mol. Cell Biol..

[58] Ohno N., Kidd G.J., Mahad D., Kiryu-Seo S., Avishai A., Komuro H., Trapp B.D. (2011). Myelination and Axonal Electrical Activity Modulate the Distribution and Motility of Mitochondria at CNS Nodes of Ranvier. J. Neurosci..

[59] Zhang Z., David G. (2016). Stimulation-induced Ca2+ influx at nodes of Ranvier in mouse peripheral motor axons. J. Physiol..

[60] Pan X., Zhou Y., Hotulainen P., Meunier F.A., Wang T. (2021). The axonal radial contractility: Structural basis underlying a new form of neural plasticity. Bioessays.

[61] Cioni J.-M., Lin J.Q., Holtermann A.V., Koppers M., Jakobs M.A.H., Azizi A., Turner-Bridger B., Shigeoka T., Franze K., Harris W.A., Holt C.E. (2019). Late Endosomes Act as mRNA Translation Platforms and Sustain Mitochondria in Axons. Cell.

[62] Liao Y.-C., Fernandopulle M.S., Wang G., Choi H., Hao L., Drerup C.M., Patel R., Qamar S., Nixon-Abell J., Shen Y. (2019). RNA Granules Hitchhike on Lysosomes for Long- Distance Transport, Using Annexin A11 as a Molecular Tether. Cell.

[63] Obara C.J., Nixon-Abell J., Moore A.S., Riccio F., Hoffman D.P., Shtengel G., Xu C.S., Schaefer K., Pasolli H.A., Masson J.-B. (2024). Motion of VAPB molecules reveals ER–mitochondria contact site subdomains. Nature.

[64] Bilsland L.G., Sahai E., Kelly G., Golding M., Greensmith L., Schiavo G. (2010). Deficits in axonal transport precede ALS symptoms in vivo. Proc. Natl. Acad. Sci..

[65] Sleigh J.N., Mattedi F., Richter S., Annuario E., Ng K., Steinmark I.E., Ivanova I., Darabán I.L., Joshi P.P., Rhymes E.R. (2024). Age-specific and compartment-dependent changes in mitochondrial homeostasis and cytoplasmic viscosity in mouse peripheral neurons. Aging Cell.

[66] Roy S. (2020). Finding order in slow axonal transport. Curr. Opin. Neurobiol..

[67] Twelvetrees A.E. (2020). The lifecycle of the neuronal microtubule transport machinery. Semin. Cell Dev. Biol..

[68] Boiko T., Van Wart A., Caldwell J.H., Levinson S.R., Trimmer J.S., Matthews G. (2003). Functional specialization of the axon initial segment by isoform-specific sodium channel targeting. J. Neurosci..

[69] Thome C., Janssen J.M., Karabulut S., Acuna C., D’Este E., Soyka S.J., Baum K., Bock M., Lehmann N., Hasegawa M. (2023). Live imaging of excitable axonal microdomains in ankyrin-G-GFP mice. Elife.

[70] Hedstrom K.L., Xu X., Ogawa Y., Frischknecht R., Seidenbecher C.I., Shrager P., Rasband M.N. (2007). Neurofascin assembles a specialized extracellular matrix at the axon initial segment. J. Cell Biol..

[71] Sheng Z.-H., Cai Q. (2012). Mitochondrial transport in neurons: impact on synaptic homeostasis and neurodegeneration. Nat. Rev. Neurosci..

[72] Sun T., Qiao H., Pan P.-Y., Chen Y., Sheng Z.-H. (2013). Motile Axonal Mitochondria Contribute to the Variability of Presynaptic Strength. Cell Rep..

[73] Sheng Z.-H. (2017). The Interplay of Axonal Energy Homeostasis and Mitochondrial Trafficking and Anchoring. Trends Cell Biol..

[74] Wang X., Schwarz T.L. (2009). The Mechanism of Ca2+-Dependent Regulation of Kinesin-Mediated Mitochondrial Motility. Cell.

[75] Gibbs K.L., Kalmar B., Rhymes E.R., Fellows A.D., Ahmed M., Whiting P., Davies C.H., Greensmith L., Schiavo G. (2018). Inhibiting p38 MAPK alpha rescues axonal retrograde transport defects in a mouse model of ALS. Cell Death Dis..

[76] Morfini G.A., Bosco D.A., Brown H., Gatto R., Kaminska A., Song Y., Molla L., Baker L., Marangoni M.N., Berth S. (2013). Inhibition of Fast Axonal Transport by Pathogenic SOD1 Involves Activation of p38 MAP Kinase. PLoS One.

[77] Sama R.R.K., Fallini C., Gatto R., McKeon J.E., Song Y., Rotunno M.S., Penaranda S., Abdurakhmanov I., Landers J.E., Morfini G. (2017). ALS-linked FUS exerts a gain of toxic function involving aberrant p38 MAPK activation. Sci. Rep..

[78] Villarroel-Campos D., Schiavo G., Lazo O.M. (2018). The many disguises of the signalling endosome. FEBS Lett..

[79] Budzinska M.I., Villarroel-Campos D., Golding M., Weston A., Collinson L., Snijders A.P., Schiavo G. (2020). PTPN23 binds the dynein adaptor BICD1 and is required for endocytic sorting of neurotrophin receptors. J. Cell Sci..

[80] Rhymes E.R., Tosolini A.P., Fellows A.D., Mahy W., McDonald N.Q., Schiavo G. (2022). Bimodal regulation of axonal transport by the GDNF-RET signalling axis in healthy and diseased motor neurons. Cell Death Dis..

[81] Rhymes E.R., Simkin R.L., Qu J., Villarroel-Campos D., Surana S., Tong Y., Shapiro R., Burgess R.W., Yang X.-L., Schiavo G., Sleigh J.N. (2024). Boosting BDNF in muscle rescues impaired axonal transport in a mouse model of DI-CMTC peripheral neuropathy. Neurobiol. Dis..

[82] Moya-Alvarado G., Tiburcio-Felix R., Ibáñez M.R., Aguirre-Soto A.A., Guerra M.V., Wu C., Mobley W.C., Perlson E., Bronfman F.C. (2023). BDNF/TrkB signaling endosomes in axons coordinate CREB/mTOR activation and protein synthesis in the cell body to induce dendritic growth in cortical neurons. Elife.

[83] Vargas J.N.S., Brown A.L., Sun K., Hagemann C., Geary B., Villarroel-Campos D., Bryce-Smith S., Zanovello M., Lombardo M., Ryandov E. (2023). BDNF controls phosphorylation and transcriptional networks governing cytoskeleton organization and axonal regeneration. bioRxiv.

[84] Zala D., Hinckelmann M.-V., Yu H., Lyra da Cunha M.M., Liot G., Cordelières F.P., Marco S., Saudou F. (2013). Vesicular Glycolysis Provides On-Board Energy for Fast Axonal Transport. Cell.

[85] Hinckelmann M.-V., Virlogeux A., Niehage C., Poujol C., Choquet D., Hoflack B., Zala D., Saudou F. (2016). Self-propelling vesicles define glycolysis as the minimal energy machinery for neuronal transport. Nat. Commun..

[86] Shigeoka T., Jung H., Jung J., Turner-Bridger B., Ohk J., Lin J.Q., Amieux P.S., Holt C.E. (2016). Dynamic Axonal Translation in Developing and Mature Visual Circuits. Cell.

[87] Vargas J.N.S., Sleigh J.N., Schiavo G. (2022). Coupling axonal mRNA transport and local translation to organelle maintenance and function. Curr. Opin. Cell Biol..

[88] Zimmermann H. (1996). Accumulation of synaptic vesicle proteins and cytoskeletal specializations at the peripheral node of Ranvier. Microsc. Res. Tech..

[89] Appeltshauser L., Linke J., Heil H.S., Karus C., Schenk J., Hemmen K., Sommer C., Doppler K., Heinze K.G. (2023). Super-resolution imaging pinpoints the periodic ultrastructure at the human node of Ranvier and its disruption in patients with polyneuropathy. Neurobiol. Dis..

[90] Eshed-Eisenbach Y., Brophy P.J., Peles E. (2023). Nodes of Ranvier in health and disease. J. Peripher. Nerv. Syst..

[91] Gong S., Zheng C., Doughty M.L., Losos K., Didkovsky N., Schambra U.B., Nowak N.J., Joyner A., Leblanc G., Hatten M.E., Heintz N. (2003). A gene expression atlas of the central nervous system based on bacterial artificial chromosomes. Nature.

[92] Misgeld T., Kerschensteiner M., Bareyre F.M., Burgess R.W., Lichtman J.W. (2007). Imaging axonal transport of mitochondria in vivo. Nat. Methods.

[93] Mallon B.S., Shick H.E., Kidd G.J., Macklin W.B. (2002). Proteolipid promoter activity distinguishes two populations of NG2-positive cells throughout neonatal cortical development. J. Neurosci..

[94] Restani L., Giribaldi F., Manich M., Bercsenyi K., Menendez G., Rossetto O., Caleo M., Schiavo G. (2012). Botulinum Neurotoxins A and E Undergo Retrograde Axonal Transport in Primary Motor Neurons. PLoS Pathog..

[95] Mohan R., Tosolini A.P., Morris R. (2014). Targeting the motor end plates in the mouse hindlimb gives access to a greater number of spinal cord motor neurons: An approach to maximize retrograde transport. Neuroscience.

[96] Mohan R., Tosolini A.P., Morris R. (2015). Intramuscular Injections Along the Motor End Plates: A Minimally Invasive Approach to Shuttle Tracers Directly into Motor Neurons. J. Vis. Exp..

[97] Gibbs K.L., Kalmar B., Sleigh J.N., Greensmith L., Schiavo G. (2016). In vivo imaging of axonal transport in murine motor and sensory neurons. J. Neurosci. Methods.

[98] Kalinski A.L., Kar A.N., Craver J., Tosolini A.P., Sleigh J.N., Lee S.J., Hawthorne A., Brito-Vargas P., Miller-Randolph S., Passino R. (2019). Deacetylation of Miro1 by HDAC6 blocks mitochondrial transport and mediates axon growth inhibition. J. Cell Biol..

