# Supplementary material for: The node of Ranvier influences the in vivo axonal transport of mitochondria and signaling endosomes
Source: iScience. 2024 Oct 11;27(11):111158. doi: 10.1016/j.isci.2024.111158 (PMC11544082; doi:10.1016/j.isci.2024.111158)
Supplement: Document S1. Figures S1–S6 [file mmc1.pdf]

iScience, Volume *27*

Supplemental information

The node of Ranvier influences the *in vivo* axonal transport of mitochondria and signaling endosomes

Andrew P. Tosolini, Federico Abatecola, Samuele Negro, James N. Sleigh, and Giampietro Schiavo

Supplemental Information

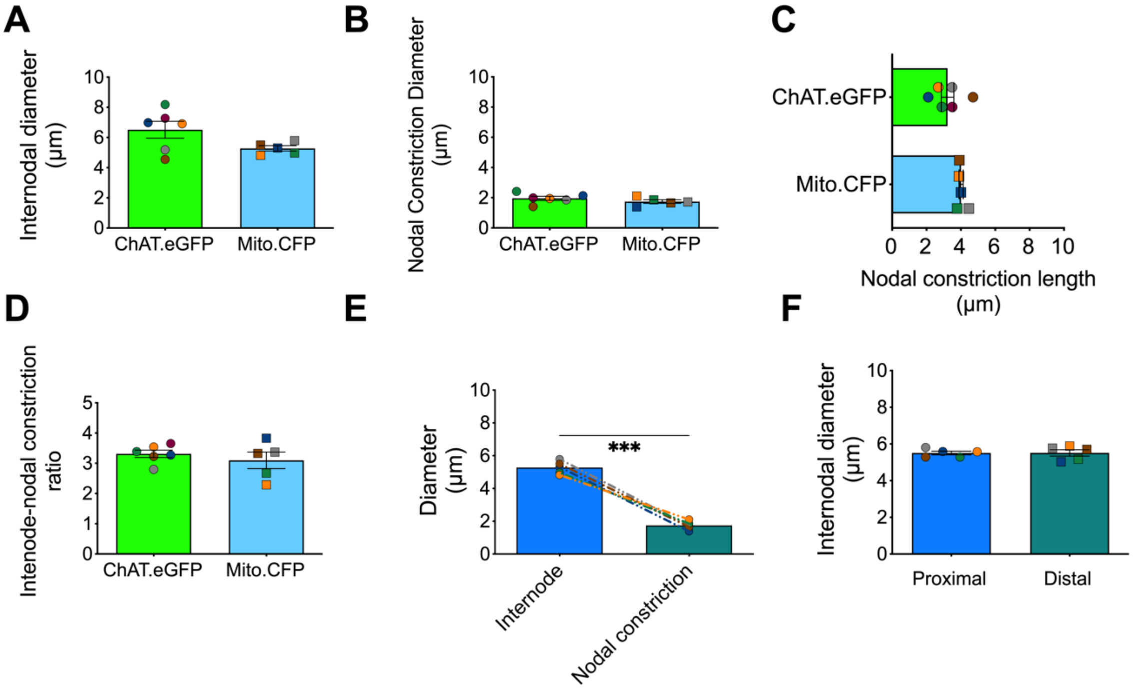

Figure S1. Nodal morphology of fast motor axons in the sciatic nerve is similar in ChAT.eGFP and Mito.CFP mice - related to Figures 1, 2 & 6. Motor axons innervating tibialis anterior (TA) muscles from both ChAT.eGFP and Mito.CFP mice display similar **A)** internodal diameters (p = 0.08, unpaired two-tailed *t*-test), **B)** nodal constriction diameters (p = 0.28, unpaired two-tailed *t*-test), **C)** nodal constriction lengths (p = 0.09, unpaired two-tailed *t*-test), and **D**) ratios of internodal to nodal diameters (p = 0.46, unpaired two-tailed *t*-test). **E**) In Mito.CFP mice, alterations in axonal diameters at the internode and nodal constriction were observed (***p = 0.0002, paired two-tailed *t*-test) and are comparable to findings from ChAT.eGFP mice (e.g., **Fig 1E**). **F**) There is no difference between the proximal and distal internodal diameters of Mito.CFP mice (p = 0.99, paired two-tailed *t*-test). For all graphs, n=5-6, means ± SEM are plotted, and the colour-coding remains consistent between animals and that of **Figure 1**.

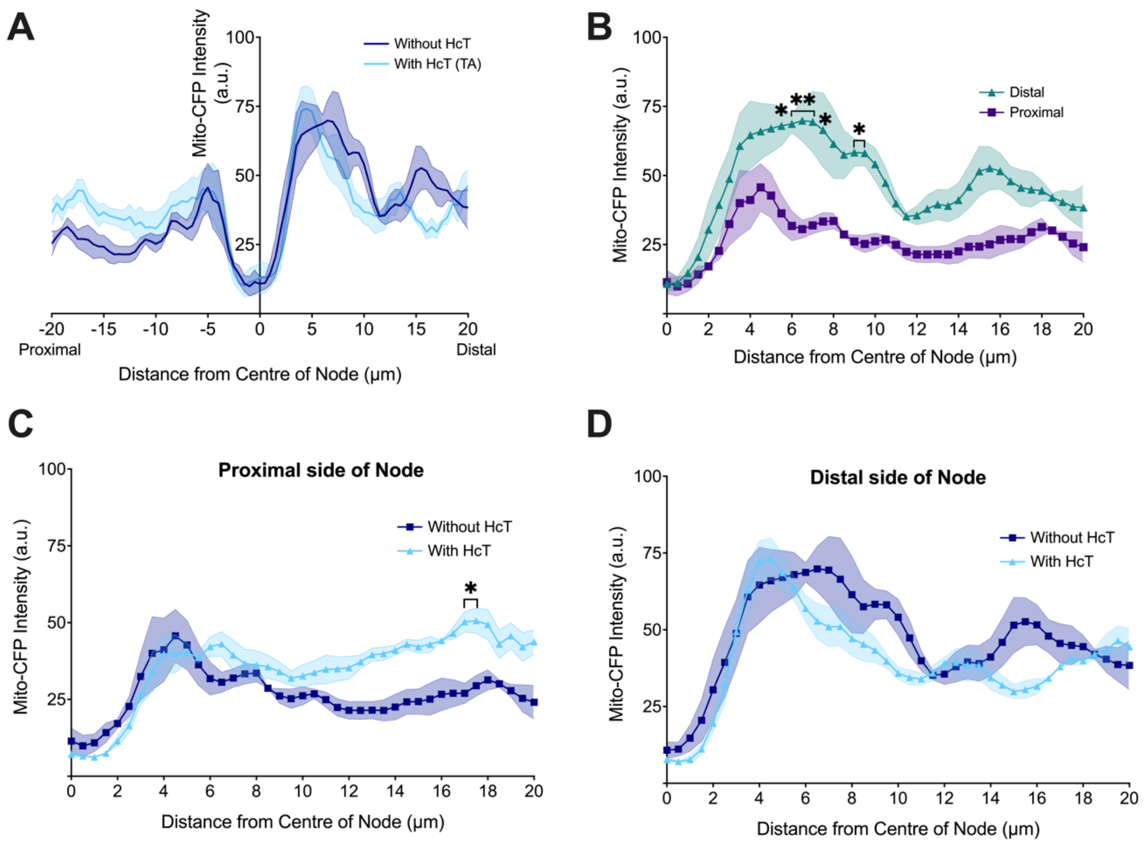

Figure S2. Mitochondrial clustering distal to the node of Ranvier is independent of HcT injection - related to Figure 2. A) In sciatic nerve axons, we observed similar patterns of fluorescence intensity and localisation of Mito.CFP labelled mitochondria in experiments with (cyan) or without (navy) intramuscular HcT injections. **B)** In mice without intramuscular injections, we observed similar increases in mean fluorescence intensities of Mito.CFP-labelled mitochondria on the distal side of the NoR. Comparing 'with HcT' (cyan) vs 'without HcT' (navy) indicates similar Mito.CFP mean fluorescence patterns on both the **C)** proximal (Axonal Location: $p < 0.001$; Mean Relative Fluorescence: $p < 0.001$; Interaction: $p < 0.001$); and **D)** distal (Axonal Location: $p < 0.001$; Mean Relative Fluorescence: $p < 0.001$; Interaction: $p = 0.7746$) sides of the NoR. For all graphs, , n=5 animals, 16 axons, means (solid line) ± SEM (dashed lines) are plotted. Data were compared by two-way ANOVA and Šídák's multiple comparisons tests (*$p < 0.05$, **$p < 0.01$).

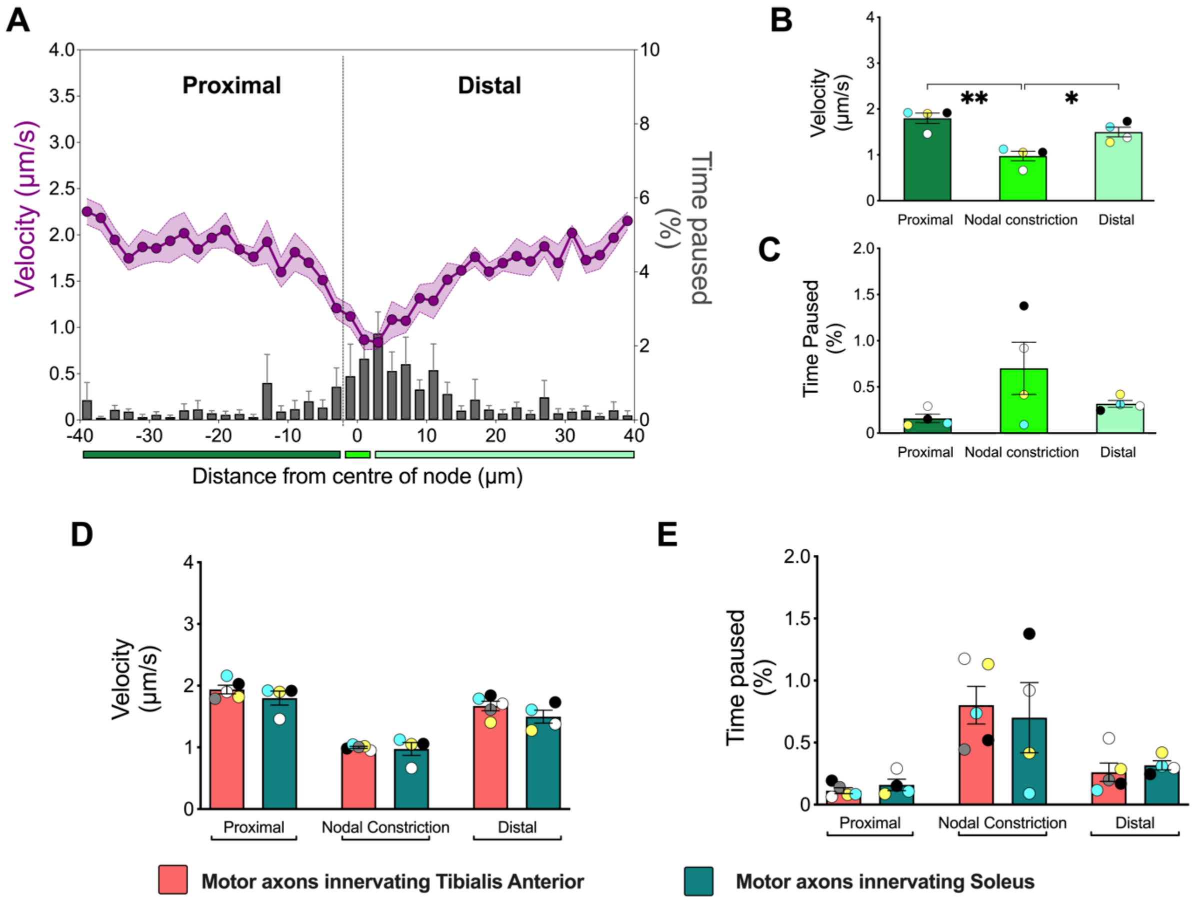

Figure S3. Signalling endosome dynamics through the node are similar between TA- and soleus-innervating motor axons – related to Figure 5. A) Retrograde axonal transport dynamics (mean moving velocity [purple] and relative frequency of mean pausing [grey bars]) of HcT-containing signalling endosomes across the nodal constriction and beyond (80 µm distance) in motor axons innervating the soleus muscle. The x-axis represents the distance from the centre of the node of Ranvier (µm) and is split into three segments: 1) *Proximal* = 38 µm of the proximal internode (dark green), 2) *Centre* = 4 µm representing the mean nodal length (as determined in **Figure 1D**; bright green), and 3) *Distal* = 38 µm of the distal internode (light green). Comparisons across the proximal internode, nodal constriction and distal internode of the **B)** mean moving velocity ($p = 0.0014$), and **C)** relative frequency of mean pausing ($p = 0.11$). Comparisons from motor axons innervating the tibialis anterior (salmon) and soleus (teal) of the **D)** mean moving velocity ($p < 0.001$) and **E)** relative frequency of mean pausing ($p = 0.0031$). Data were compared by an ordinary one-way ANOVA, followed by Holm-Šídák's multiple comparisons test (*$p < 0.05$, **$p < 0.01$). For all graphs, means (solid line) ± SEM (dashed lines/error bars) are plotted, n=4 (soleus) and n=5 (tibialis anterior) Mito.CFP mice.

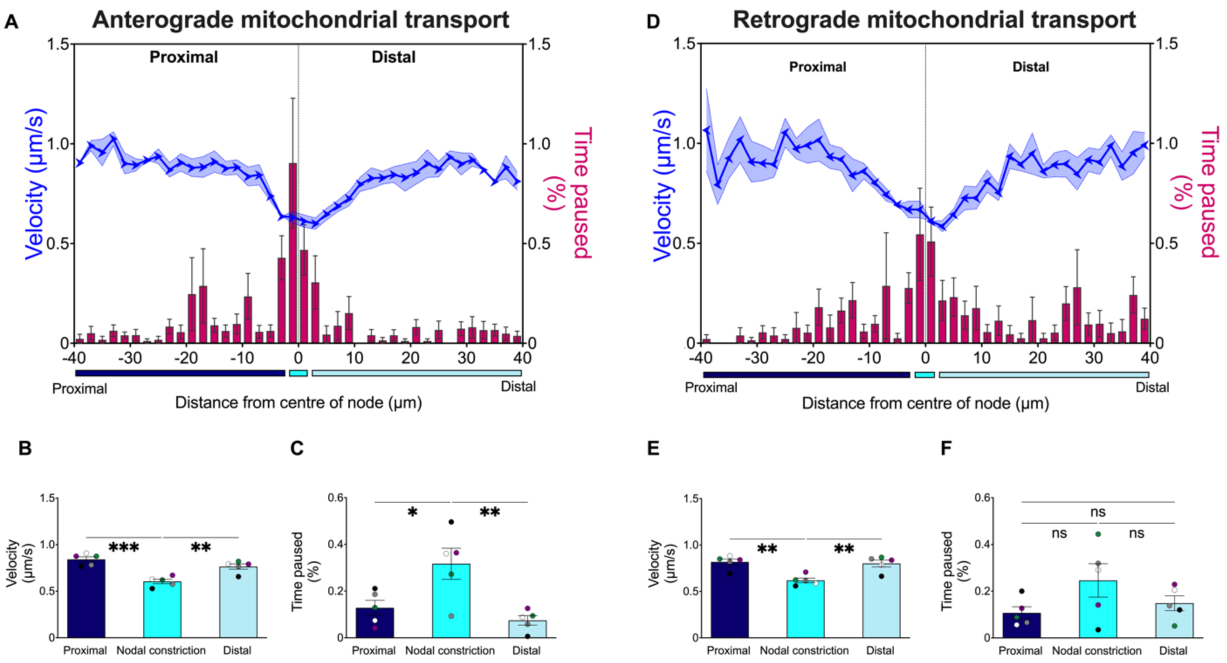

Figure S4. At the nodal constriction, anterogradely moving mitochondria slow down and pause more, whereas retrogradely moving mitochondria display reduced velocity without changes in pausing – related to Figure 6. *In vivo* mitochondrial **A)** anterograde and **D)** retrograde transport dynamics (mean moving velocity [blue arrowheads] and relative frequency of mean pausing [pink bars]) across the nodal constriction and beyond (80 μm distance) in axons innervating the tibialis anterior muscle. The x-axis represents the distance from the centre of the node of Ranvier (μm) and is split into three segments: 1) *Proximal* = 38 μm of the proximal internode (navy); 2) *Centre* = 4 μm representing the mean nodal length (as determined in **Figure 1D**; cyan); and 3) *Distal* = 38 μm of the distal internode (light blue). Comparisons of anterogradely moving mitochondria across the proximal internode, nodal constriction and distal internode of the **B)** mean moving velocity ($p = 0.001$), and **C)** relative frequency of mean time paused ($p = 0.0054$). Comparisons of retrogradely moving mitochondria across the proximal internode, nodal constriction and distal internode of the **E)** mean moving velocity ($p = 0.014$), and **F)** relative frequency of mean time paused ($p = 0.149$). Data were compared by one-way ANOVA, followed by Holm-Šídák's multiple comparisons test. (*p <0.05, **p <0.01, ***p <0.001, *ns* not significant). Means (solid line) ± SEM (dashed lines/error bars) are plotted for all graphs. n=5 Mito.CFP mice.

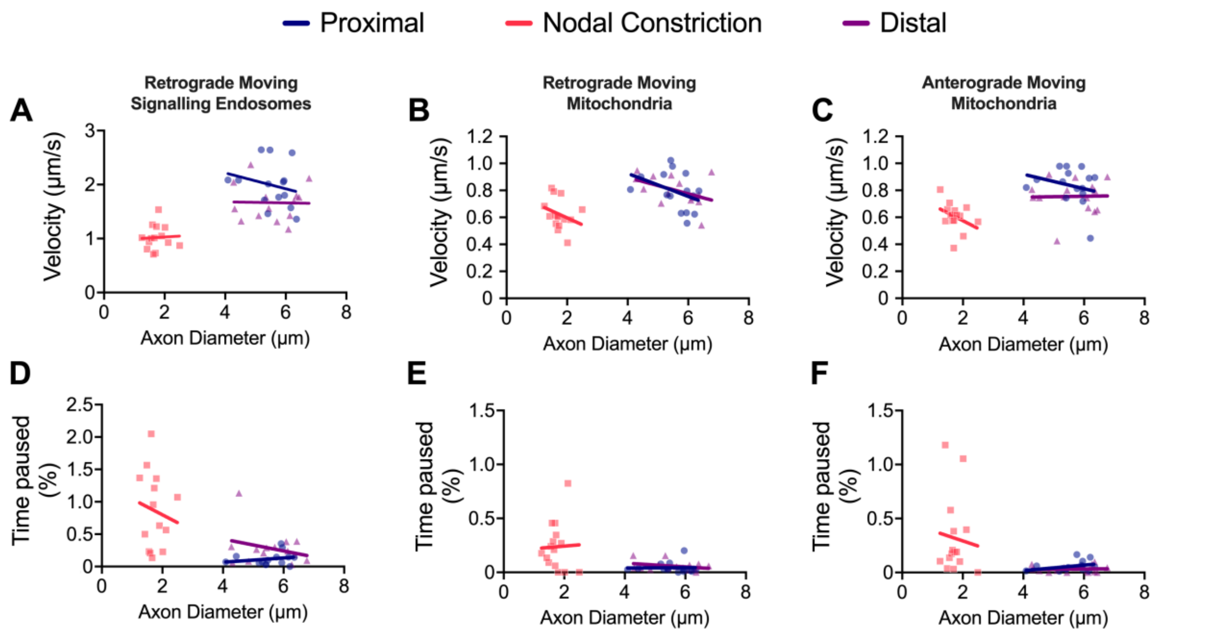

Figure S5. Axonal sub-domain width does not correlate with transport speed nor pausing. Axon diameter and velocity are not correlated for **A)** retrogradely moving signalling endosomes (Proximal: r = -0.23; p = 0.43; Nodal Constriction: r = 0.05; p = 0.87; Distal: r = -0.02; p = 0.94), **B)** retrogradely moving mitochondria (Proximal: r = -0.39; p = 0.15; Nodal Constriction: r = -0.28; p = 0.31; Distal: r = -0.46; p = 0.08), nor **C)** anterogradely moving mitochondria (Proximal: r = -0.25; p = 0.36; Nodal Constriction: r = -0.35; p = 0.20; Distal: r = 0.03; p = 0.94). Time paused and axon width are also not correlated for **D)** retrogradely moving signalling endosomes (Proximal: r = 0.22; p = 0.46; Nodal Constriction: r = -0.13; p = 0.66; Distal: r = -0.28; p = 0.33), **E)** retrogradely moving mitochondria (Proximal: r = 0.02; p = 0.93; Nodal Constriction: r = 0.03; p = 0.91; Distal: r = -0.33; p = 0.23), nor **F)** anterogradely moving mitochondria (Proximal: r = 0.35; p = 0.20; Nodal Constriction: r = -0.08; p = 0.78; Distal: r = 0.06; p = 0.83). For all graphs, the line of best fit and individual datapoints are plotted for the proximal internode (navy; circles), nodal constriction (salmon; squares) and distal internode (purple; triangles). Two-tailed, Pearson correlation coefficients (r) were computed. n=15 axons from n=5 Mito.CFP mice.

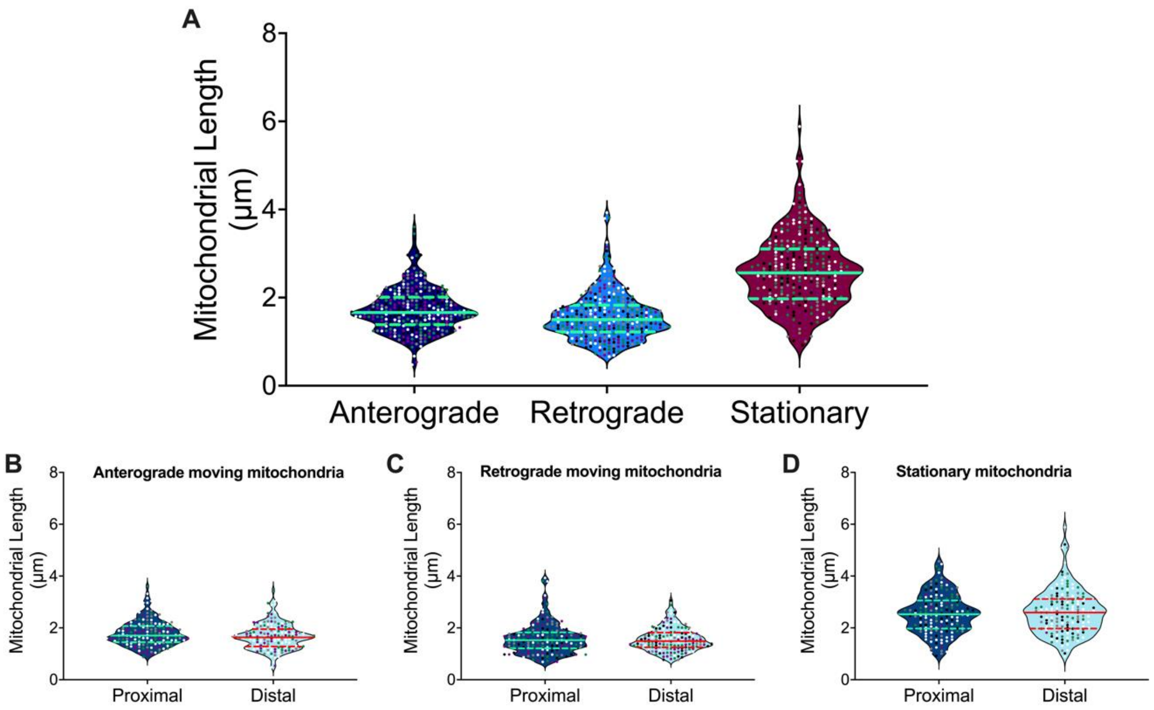

Figure S6. Stationary mitochondria are longer than motile mitochondria regardless of axonal sub-domain – linked with Figure 6G. A) Individual lengths for the anterogradely moving (n=308 mitochondria; navy), retrogradely moving (n=311 mitochondria; blue) and stationary mitochondria (n=300 mitochondria; maroon) of the combined proximal (navy) and distal (light blue) internode region. Individual lengths from the proximal and distal internodes of **B)** anterogradely moving mitochondria (proximal: n=159 mitochondria; distal: n=152 mitochondria), **C)** retrogradely moving mitochondria and **D)** stationary mitochondria (proximal: n=151 mitochondria; distal: n=149 mitochondria). n=5 Mito.CFP mice. Colour coding of individual data points represents an individual animal, and match their corresponding graph in Fig. 6G. The turquoise/red lines represent the mean, and the dashed turquoise/red lines represent the upper and lower quartiles.
